# Acoustic monitoring reveals spatiotemporal occurrence of Nathusius’ pipistrelle at the southern North Sea during autumn migration

**DOI:** 10.1007/s10661-023-11590-2

**Published:** 2023-08-02

**Authors:** Sander Lagerveld, Tony Wilkes, Marinka E. B. van Puijenbroek, Bart C. A. Noort, Steve C. V. Geelhoed

**Affiliations:** Den Helder, Wageningen University & Research, Ankerpark 27, 1781 AG den Helder, The Netherlands

**Keywords:** Bat migration, Offshore wind farms, Offshore migration, Offshore distribution, *Pipistrellus nathusii*

## Abstract

Seasonal movements between the summer and winter areas are a widespread phenomenon in bats So far, most information on the migration ecology of bats has been obtained by studies in terrestrial habitats, whereas scientific knowledge on migration over sea is scarce. We performed continuous ultrasonic acoustic monitoring at 13 locations in the southern North Sea during four consecutive years (2017–2020) and analysed the spatiotemporal occurrence of Nathusius’ pipistrelle *Pipistrellus nathusii* during autumn migration in relation to weather parameters and lunar phase. Our analysis showed that the main autumn migration of Nathusius’ pipistrelle at the southern North Sea occurs from mid-August until late October and most bats within the study area occur off the Noord Holland coast. North Sea crossings frequently last longer than one night; the day is spent roosting at an offshore structure. The strongest migration occurs during nights with tailwinds from the east-northeast, but bats are also recorded offshore with low to moderate headwinds or crosswinds. Bat presence decreased between the full moon and the last quarter and increased just before the new moon. Finally, our observations show that the occurrence of bats at sea was reduced in 2020 in comparison to the previous years. The results of this study show clear spatiotemporal patterns of migratory bat occurrence at the southern North Sea. The spatial distribution can be used in spatial planning of future offshore wind farms, whereas the temporal occurrence and environmental factors that shape offshore migration can be used to develop mitigation measures to reduce the number of bat fatalities.

**Supplementary Information:**

The online version contains supplementary material available at 10.1007/s10661-023-11590-2.

## Introduction

Migration between summer and winter areas is a widespread phenomenon in bats (Ciechanowski et al., 2016; Fleming & Eby, 2003; Hutterer et al., 2005; Krauel et al., 2013; Popa-Lisseanu & Voigt, 2009). In temperate areas, migratory species exhibit regional (typically 100–500 km) or long-distance (> 1000 km) seasonal movements between breeding and wintering areas. Partial migration also occurs, in which some populations perform seasonal migrations while other populations remain sedentary (Krauel et al., 2013).

Nathusius pipistrelle *Pipistrellus nathusii* is an example of such a partial migrant. The species’ main breeding areas are found in central and eastern Europe, of which populations in central Europe are sedentary or migrate over short distances (Sachanowicz et al., 2019), and eastern populations perform long-distance migrations. The longest known migration distances in autumn are from Latvia to Spain and from Russia to France, respectively, 2224 and 2486 km (Alcalde et al., 2021; Vasenkov et al., 2022). After the breeding season, females and their offspring migrate predominantly to their wintering areas in southern and western Europe, including the United Kingdom (Russ et al., 2001; National Nathusius’ Pipistrelle Project, 2022). Ringing recoveries from the United Kingdom have confirmed that most individuals followed an east-northeast (ENE)-west-southwest (WSW) route during their migration to and from the breeding areas in north-eastern Europe (National Nathusius’ Pipistrelle Project, 2022). During the post-breeding migration, mating takes place along the way, where adult mates advertise to attract passing females (Jahelkova & Horacek, 2011). After the mating season, some males migrate to wintering areas at lower latitudes, while others winter in the same area (Pētersons, 2004; Sachanowicz et al., 2019).

Most of the species’ seasonal movements occur over terrestrial habitats, but migration also takes place over sea (Ahlén et al., 2009; Boshamer & Bekker, 2008; Brabant et al., 2021; Hüppop & Hill, 2016; Lagerveld et al., 2014, 2021; Rydell et al., 2014). During migration over land, Nathusius’ pipistrelle often uses guiding landscape structures like river valleys (Furmankiewicz & Kucharska, 2009) or coasts (Ijäs et al., 2017; Pētersons, 2004). Bats aggregate along the coast prior to their departure over sea, and higher levels of bat activity have been recorded at sea close to known departure locations of migrant individuals in southern Sweden (Ahlén et al., 2009). Yet, it is unknown whether distinct spatial patterns exist further away from the coast, or that bats migrate over sea in a broad front like many songbirds do (Lack, 1963a, b).

At the North Sea, Nathusius’ pipistrelle is the most frequently observed bat species (Boshamer & Bekker, 2008; Brabant et al., 2021; Hüppop & Hill, 2016; Lagerveld et al., 2014, 2021). An analysis of the species’ autumn occurrence in 2012–2016 at three offshore wind farms off the Dutch coast revealed that most migration over sea occurs during easterly winds with a distinct peak at ENE, wind speeds < 5 m/s and temperatures > 15 °C (Lagerveld et al., 2021). Off the Belgian coast, a relationship with light easterly winds and higher temperatures was also found, in addition to a relatively high atmospheric pressure (Brabant et al., 2021). In the German Bight, however, most bats are recorded in overcast conditions with precipitation during southerly winds (Hüppop & Hill, 2016).

During migration over sea, bats might encounter wind farms, which on land have reportedly caused significant numbers of bat fatalities (Arnett et al., 2008; Bach & Rahmel, 2004; Baerwald et al., 2009; Cryan et al., 2014; Grodsky et al., 2011; Hayes, 2013; Kunz et al., 2007; Voigt et al., 2015) due to collisions and, in a lesser extent, barotrauma (Lawson et al., 2020; Rollins et al., 2012). Most fatalities concern migratory species and occur in late summer and early autumn (Arnett et al., 2008; Bach & Rahmel, 2004; Baerwald et al., 2009; Cryan et al., 2014; Grodsky et al., 2011; Hayes, 2013; Kunz et al., 2007; Voigt et al., 2015). The location of wind farms is an important determinant of the mortality rate (Rydell et al., 2010). Next to the location choice, the number of fatalities can be reduced by operational measures. Effective mitigation has been achieved by limiting the production time of wind turbines by switching them off during periods when bats are most active (Adams et al., 2021; Arnett et al., 2011).

As bats behave similarly around offshore wind turbines compared to wind turbines on land (Ahlén et al., 2009), wind farm-induced mortality is likely to occur at sea. Knowledge on the occurrence and distribution of migratory bats at sea is, therefore, highly needed, given the prospected extensive growth for offshore wind development in the next decades in the North Sea (European Commission, 2020). To gain this knowledge, we performed continuous ultrasonic acoustic monitoring at 13 locations in the southern North Sea during two to four consecutive years. We subsequently analysed the offshore occurrence of Nathusius pipistrelle in space and time during autumn migration in relation to weather parameters (wind, atmospheric pressure, cloud cover and precipitation) and the lunar phase.

## Methods

### Study area

**Fig. 1:**
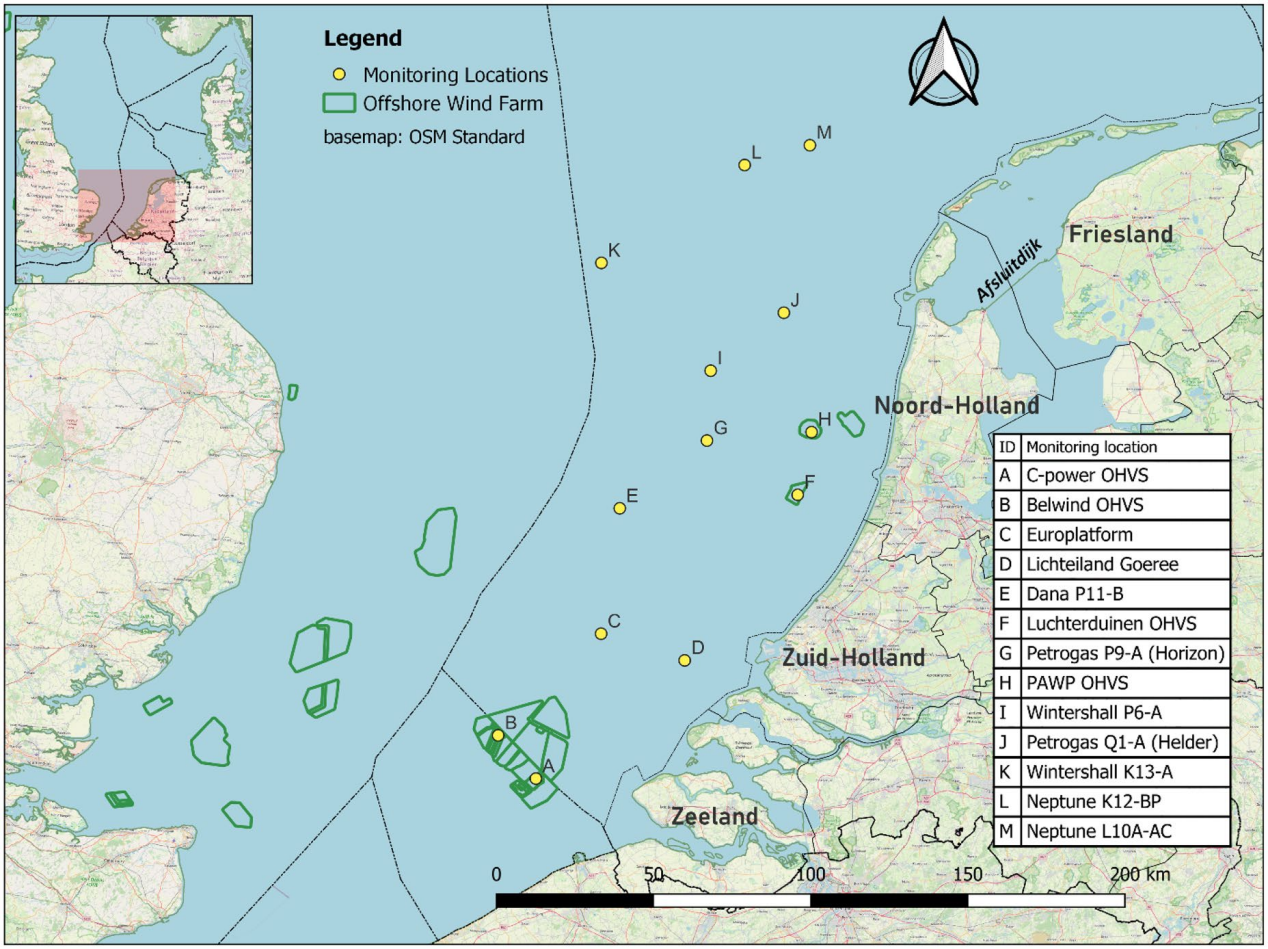
Acoustic monitoring network

Acoustic monitoring was conducted at 13 locations in the southern North Sea off the Dutch west coast (Fig. 1). The monitoring equipment was installed at offshore platforms, including four offshore high voltage stations (OHVS) in wind farms (C-Power, Belwind, Luchterduinen, and PAWP), two measurement platforms (Europlatform and Lichteiland Goeree) and seven gas production platforms (Dana P11-B, Petrogas P9-A, Wintershall P6-A, Petrogas Q1-A, Wintershall K13-A, Neptune K12-BP and Neptune L10A-AC). The monitoring periods per location per year are shown in Table 1.

**Table 1:** Annual monitoring effort per location

ID	Monitoring location	2017	2018	2019	2020
A	C-power OHVS		28/02–31/12	01/01–31/12	21/06–27/07 10/09–19/09
B	Belwind OHVS	02/08–26/12	01/03–31/12	01/01–31/12	01/01–30/09
C	Europlatform		06/03–31/12	01/01–31/12	18/02–17/05 30/07–25/09
D	Lichteiland Goeree	27/09–31/12	06/03–18/11	09/05–31/12	24/06–30/10
E	Dana P11-B			25/02–27/06 11/07–31/12	15/02–06/06 23/07–31/12
F	Luchterduinen OHVS		04/04–18/07 15/08–31/12	01/01–31/12	06/04–12/06 04/08–03/09
G	Petrogas P9-A (Horizon)		15/11–31/12	01/01–31/12	01/01–08/06 25/06–07/07 26/07–16/11
H	PAWP OHVS	02/08–31/12	13/01–31/12	01/01–18/10 22/11–31/12	01/01–07/02 04/03–31/12
I	Wintershall P6-A	31/10–31/12	11/03–21/12	01/01–31/12	01/01–04/06 13/07–31/12
J	Petrogas Q1-A (Helder)		11/09–31/12	01/01–31/12	01/01–31/12
K	Wintershall K13-A			01/01–22/08 19/09–27/09 01/12–31/12	04/08–16/12
L	Neptune K12-BP	26/07–11/12	24/01–31/12	01/01–31/12	18/03–31/05 22/09–09/11
M	Neptune L10A-AC	18/08–31/12	01/01–31/12	01/01–31/12	21/03–08/04 23/09–13/11

#### Acoustic monitoring

Acoustic bat activity was monitored with an Avisoft - UltraSoundGate 116Hnbm in combination with a horizontal-mounted omnidirectional electret ultrasound microphone FG-DT50, at heights between 15 and 33 m above sea level (average 21 m). The microphone was enclosed in a waterproof box and connected to the soundgate in an on-site computer room. Microphones were replaced twice a year, late February/early March and late July/early August, and subsequently recalibrated by the manufacturer, Avisoft Bioacoustics. See online resource 1 for details on the monitoring locations and Table 2 for the settings of the acoustic monitoring equipment.

**Table 2:** Settings of the Avisoft - UltraSoundGate 116Hnbm

**Parameter**	**Value**	**Parameter**	**Value**
Pre-trigger	0.1 s	Buffer	0.064 s
Hold tm	0.8 s	Bat call filter	Enabled
Duration	> 0 s	Accept monotonic structures	Enabled
Syllable	> 0 s	Min sweep rate FM	− 20 kHz/ms
Reject wind/rain	Enabled	Min sweep rate CF	− 3 kHz/ms
Trigger event level	0.501%	Max sweep rate FM	− 1 kHz/ms
Trigger event range	15–100 kHz	Max sweep rate CF	2 kHz/ms
Sampling rate	250,000 Hz	Min duration FM	1 ms
Format	16 bit	Min duration CF	2 ms

We used the cross-correlation function of Avisoft SASlab Pro, version 5.2.14 (Avisoft bioacoustics), to extract the recordings with bat calls from the raw monitoring data. Reference calls for the cross-correlation included recordings of Nathusius’ pipistrelle (*n* = 8), Common pipistrelle *P. pipistrellus* (*n* = 4), Pond bat *Myotis dasycneme* (*n* = 4), Common noctule *Nyctalus noctula* (*n* = 18), Serotine bat *Eptesicus* *serotinus* (*n* = 5) and the Nyctaloid group (*n* = 31, including the genera *Nyctalus*, *Vespertilio*, *Eptesicus*). Subsequently, all recordings with bat calls were assessed individually and identified to the lowest taxonomic level as possible, using the criteria provided by Barataud (2016). Feeding buzzes were identified by visually checking spectrograms of the recordings (cf Barataud, 2016) and verified acoustically by listening to the recordings.

We only included the acoustic data of Nathusius’ pipistrelle in the analysis as this is the most frequently recorded species at sea (92% of the data). We used positive minutes i.e. minutes with at least one bat recording, to visualise the monitoring results. Date-time plots were made with ggplot2 (Wickham, 2016), showing positive minutes throughout the season and the night.

#### Data preparation

Hourly offshore weather data of eight offshore weather stations (203, 204, 207, 211, 212, 252, 321, 320) was obtained from the Royal Dutch Meteorological Institute (KNMI, 2021). We used the following weather variables: wind direction averaged over 10 min, wind speed averaged over 10 min measured at an altitude of 10 m above sea level, temperature at 1.5 m height, atmospheric pressure at sea level, cloud cover in octants and precipitation (binary, either 0 or 1). Cloud class 9 (sky invisible) and wind directions with values of 0 (no wind) and 990 (variable direction) were regarded as missing values.

For each monitoring location, we used data of the nearest weather station. If values were missing, we imputed the data of the two closest weather stations. We averaged the temperature, atmospheric pressure, cloud cover and precipitation for each night. The atmospheric pressure change was calculated based on the average atmospheric pressure from a particular night minus the average atmospheric pressure from the previous night. In order to obtain an average wind speed per night and an average wind direction per night, we decomposed the hourly wind direction and speed values into “North” and “East” vector components. The vector components were averaged per night and then were used to calculate the average wind speed using the Pythagorean theorem. The angle of the vectors was used to calculate the average wind direction.

The main migration direction in autumn off the Dutch coast likely runs from ENE to WSW (Lagerveld et al., 2021; National Nathusius’ Pipistrelle Project, 2022). We used this direction to derive the average tailwind (Eq. 1) and crosswind (Eq. 2) components per night:

1 $$\mathrm{Tailwind}\;\mathrm{component}=\mathrm{COS}\ (\mathrm{wind}\;\mathrm{direction}\;\lbrack\mathrm{rad}\rbrack-\mathrm{migration}\;\mathrm{direction}\;\lbrack\mathrm{rad}\rbrack)\times\mathrm{windspeed}$$

2 $$\mathrm{Crosswind}\;\mathrm{component}=\mathrm{SIN}\;(\mathrm{wind}\;\mathrm{direction}\;\lbrack\mathrm{rad}\rbrack-\mathrm{migration}\;\mathrm{direction}\;\lbrack\mathrm{rad}\rbrack)\times\mathrm{windspeed}$$

Positive values of the tailwind component indicate supportive wind conditions in the migration direction, whereas negative values indicate headwind. The crosswind component is the wind vector perpendicular to the migration direction. Positive values of the crosswind component indicate wind from the north–north-west (NNW), and negative values indicate wind from the south-south-east (SSE).

In addition to the weather data, we included night in the year, year, latitude, longitude and lunar phase as covariates. The lunar phase was calculated using the R-package lunar (Lazaridis, 2014). Table 3 summarises the covariates included in the analysis.

**Table 3:** Covariates used in the analysis

Covariate	Definition
Night in year	Each night starts at 16:00 UTC on day 1 and continues to 16:00 UTC on day 2 1 January starts with night number 1
Year	Calendar year
Tailwind	The nightly averaged wind vector from ENE to WSW (m/s)
Crosswind	Average nightly wind vector from NNW to SSE (m/s)
Atmospheric pressure	Average nightly pressure (hPa)
Atmospheric pressure change	The change in atmospheric pressure compared to the previous night (hPa)
Temperature	Average nightly temperature (°C)
Cloud class	Average nightly cloud class (octants)
Rain	Average nightly proportion of hours with rain (%)
Lunar phase	The period of the lunar cycle is a lunar month (29.53 days) divided into 360°
Longitude	Longitude of the monitoring location
Latitude	Latitude of the monitoring location

All calculations were done in R version 4.1.2 (R Core Team, 2020) and R studio version 2021.09.2 (R Studio Team, 2021), primarily using the R-packages tidyverse (Wickham et al., 2019), lubridate (Grölemund & Wickham, 2011), data.table (Dowle & Srinivasan, 2021), matlib (Friendly et al., 2021), gstat (Gräler et al., 2016; Pebesma, 2004), sf (Pebesma, 2018), suncalc (Thieurmel & Elmarhraoui 2019) and vmstools (Hintzen et al., 2017).

#### Statistical analysis

A Bernoulli Generalized Additive Model (GAM) with a logit link function was used to model bat presence per night as a function of the covariates, using the mgcv R-package (Wood, 2017). Due to the limited number of nights with bat activity in spring (133 nights) and the absence of bat activity during winter and early summer, we limited the analysis to autumn from night number 230 (17 or 18 August, depending on the year) till night number 321 (16 or 17 November, depending on the year). The time frame considered consisted of 3255 monitoring nights in total, of which bats were recorded on 251 nights (7.7% of the data).

The correlation and linearity between the numerical covariates were assessed with a correlogram and pairs plot, using the R-packages corrgram (Wright, 2021) and GGally (Schloerke et al., 2021), respectively. Night in year appeared to be collinear with temperature, and therefore, the latter covariate was excluded from the analysis. The relationships between the response variable and the continuous covariates were checked graphically.

Longitude and latitude were entered together in a tensor product smoother to model spatial (auto)correlation. Night number was entered as a low-rank thin-plate smoother to model the seasonal-temporal pattern. Atmospheric pressure and precipitation were entered as linear covariates. Cloud class, pressure change, tailwind and crosswind were entered as low-rank thin-plate smoothers. Lunar phase was entered as a cyclic cubic regression smoother with boundary knots at 0 and 360 degrees, and the year was entered as a categorical covariate. The linear covariates were centred when applicable. The emmeans R-package (Length, 2022) was used to aid the factor level comparisons. The Bernoulli GAM model was inclined to predominately predict zeros, due to the zero-inflation. The specificity (true negative rate) was found to be very high (nearly 1), but the sensitivity (true positive rate) was very low. By adding a small positive constant to the linear predictor after the model was fit, one can sacrifice a little bit of specificity to increase the sensitivity. This post-model constant is referred to as $\alpha$. Values between 0 and 2, with increments of 0.025, were tested for $\alpha$, and the value for $\alpha$ was chosen to optimise both sensitivity and specificity and minimise the 0/1-loss, leading to $\alpha =1.95$.

Thus, the model can be formulated as follows (Eq. 3 and Eq. 4):

3 $$Y \sim Bernoulli\;(p)$$

4 $$logit (p)\sim Intercept + Covariates + \alpha$$

where *Y* is the response variable (0 = no bat activity recorded in a night, 1 = bat activity recorded in a night).

Residual diagnostic plots using the Dunn-Smyth residuals (Dunn & Smyth, 1996) were made to check for violations of the model assumptions. Some of these plots were made using the R-package DescTools (Signorell et al., 2021). In order to visualise the effects of individual covariates, predictor effect plots were made, using ggplot2 (Wickham, 2016).

## Results

### Acoustic monitoring

**Fig. 2:**
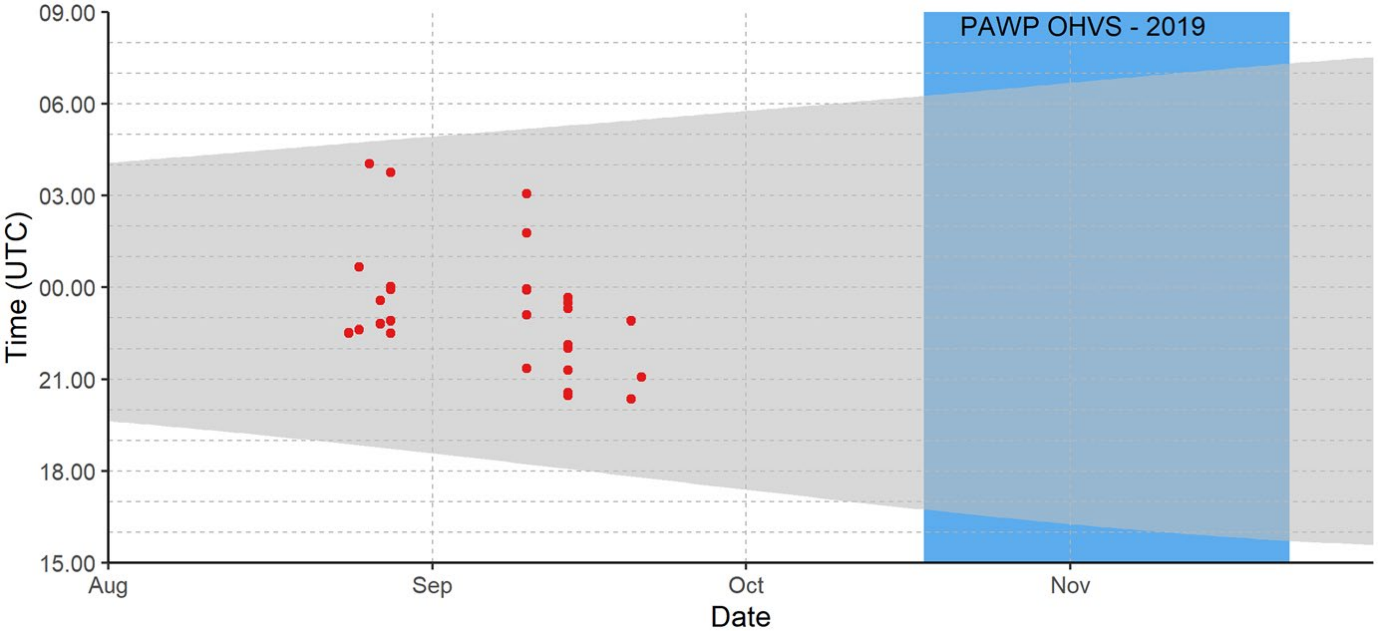
Bat activity of Nathusius’ pipistrelle autumn 2019 at PAWP OHVS, 25 km from shore. Dots are positive minutes with acoustic activity throughout the night (time interval between sunset and sunrise is represented by grey) and throughout the season. The effective monitoring period is indicated by a white background, whereas the blue background indicates no monitoring

**Fig. 3:**
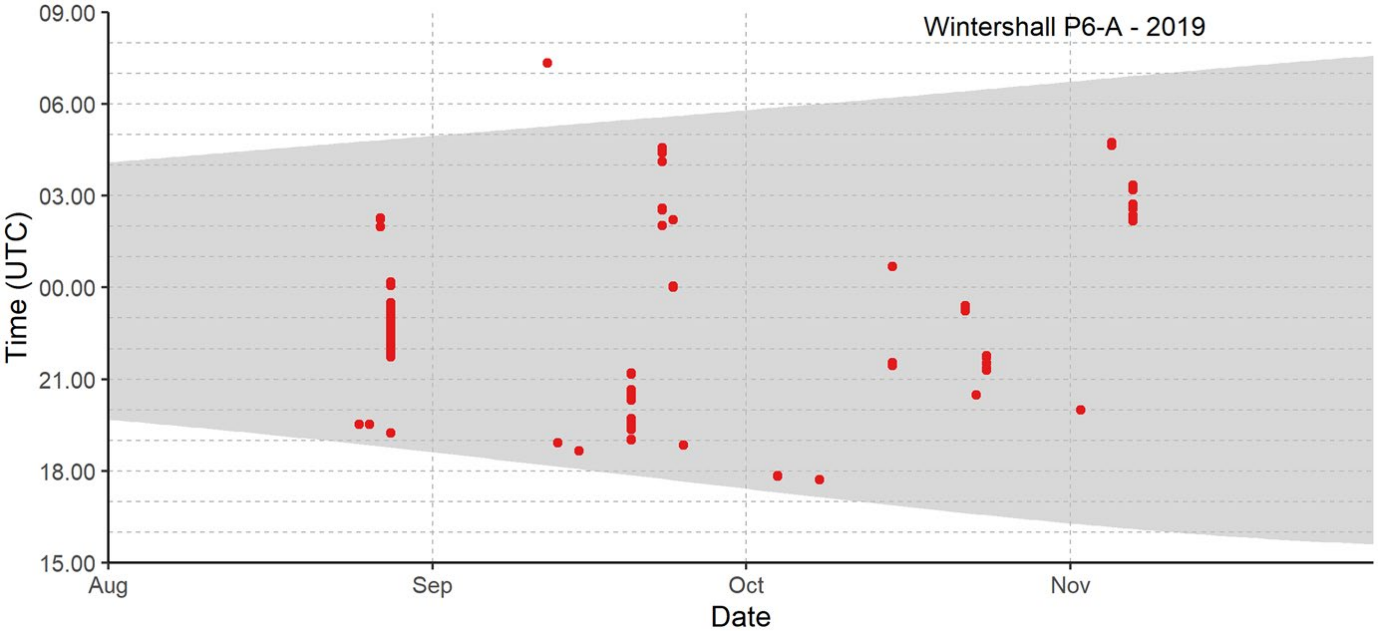
Bat activity of Nathusius’ pipistrelle autumn 2019 at Wintershall P6-A, 60 km from shore. See Fig. 2 for explanation

Nathusius’ pipistrelle has been recorded at all monitoring locations (see online resource 2), with a total of 2147 recordings. Of these, 168 contained feeding buzzes (7.8% of the recordings), which were recorded at 8 out of 13 monitoring locations. Examples of recorded bat activity at sea are shown in Figs. 2 and 3. At PAWP OHVS (25 km from shore), bat activity is recorded 2 h after sunset onwards, whereas at Wintershall P6-A (60 km from shore) bats are recorded throughout the night, including individuals which are present just after sunset. Most activity is recorded during the night, but sometimes also during daylight hours. At Wintershall P6-A, an individual arrived more than 2 h after sunrise on 12 September 2019 and (likely the same individual) was recorded again the same evening just after sunset.

### Modelled occurrence

**Fig. 4:**
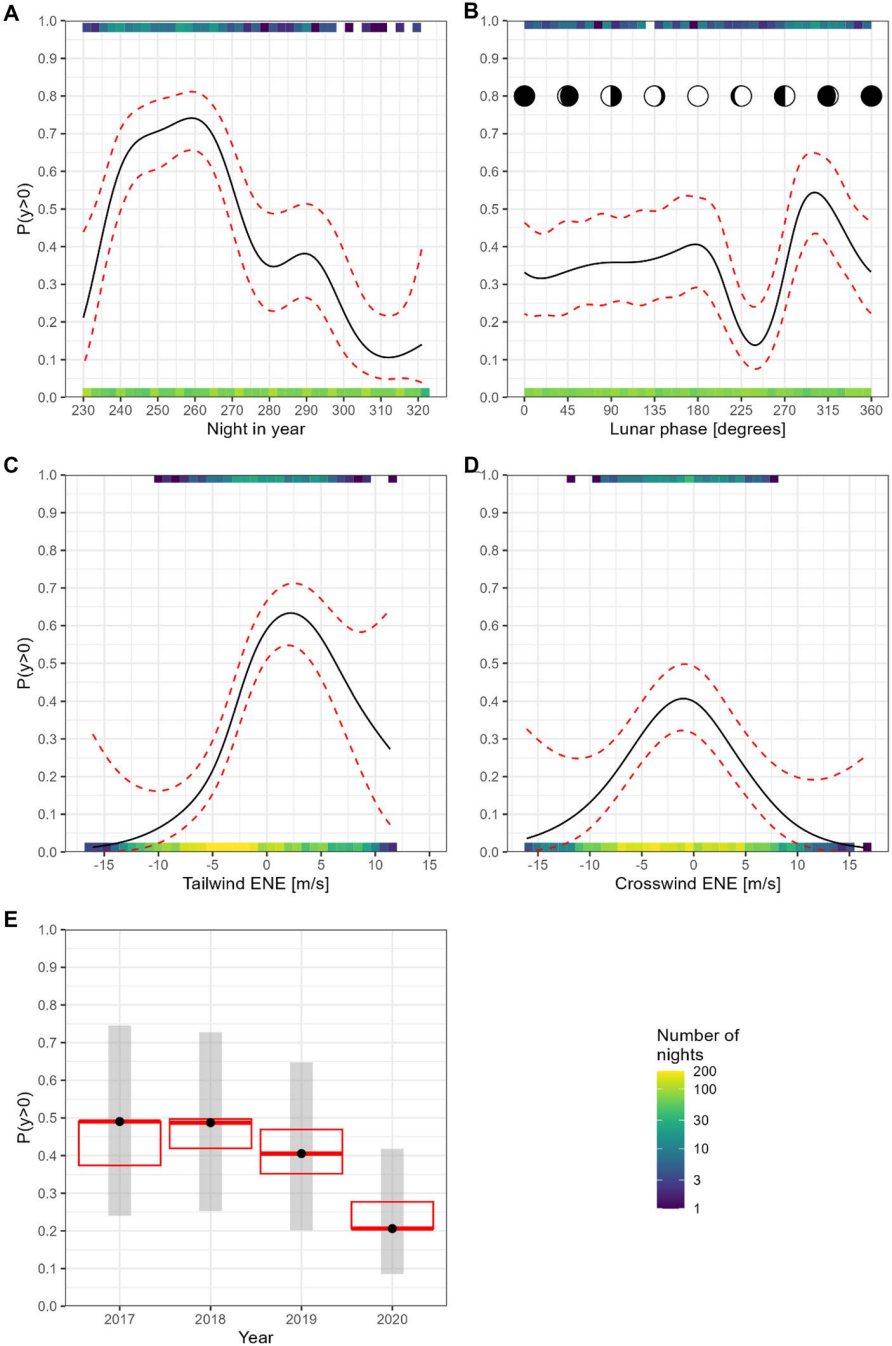
Predictor effect plots of the covariate. **A** night in year: 230 = mid-August and 320 = mid-November; **B** lunar phase: 0 and 360 degrees represent new moon, 90 degrees first quarter, 180 degrees full moon and 270 degrees third quarter; **C** tailwind: positive values indicate supportive wind conditions from ENE, whereas negative values indicate headwind from WSW; **D** crosswind: positive values indicate wind from the NNW and negative values indicate wind from SSE; and **E** calendar year. In plots A–D, the solid lines represent the expected values of the predicted probability of bat presence (on the *y*-axis), and the red dashed lines indicate the 95% confidence interval of the expected values. In plot E, the black points indicate the predicted probability of bat presence, the grey shades indicate the 95% confidence intervals of the means, and the red crossbars indicate the (Tukey-corrected) confidence intervals for pairwise differences. In all plots, only the term of interest varies, while the spatiotemporal effects are ignored (they average out to zero), and all other covariates are fixed at their mean values

**Fig. 5:**
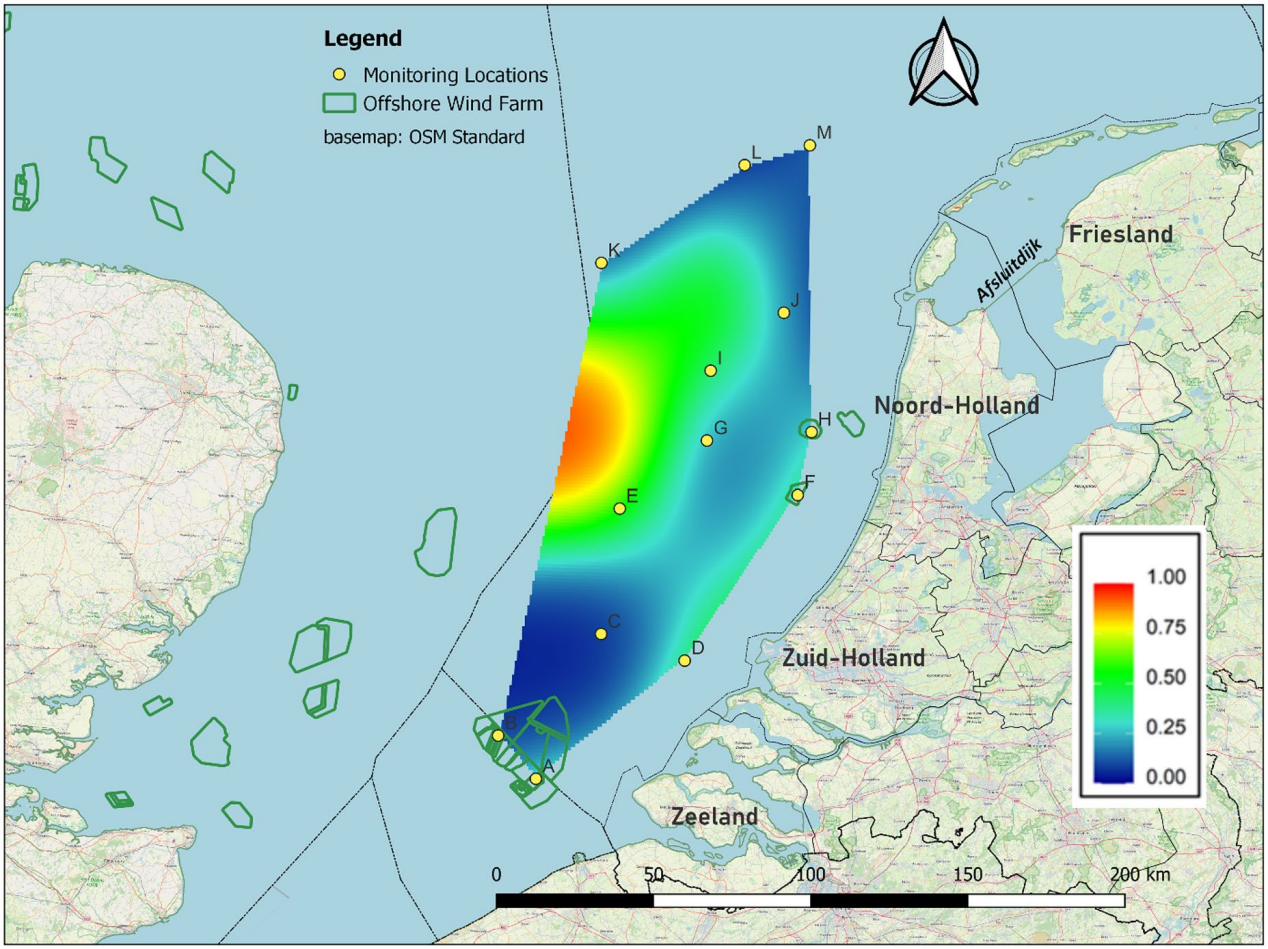
Predictor effect plot of the spatial smoother for longitude and latitude. The colour indicates the average predicted probability of presence per night, while all other covariates are fixed at their mean values

The output of the analysis can be found in online resource 3. Coefficient plots of the smoothers showed that night in year, lunar phase, tailwind and crosswind are important predictors for bat activity at sea. Cloud cover was not important, and atmospheric pressure change was of minor importance (almost significant). The tensor smoother for longitude and latitude showed that areas in the west had a higher probability of bat presence, while areas in the south, east and north had a lower probability (within the boundaries of the monitoring locations). The linear covariate atmospheric pressure proved to be not important, and precipitation was of minor importance. A significant difference was found between the year 2020 and the previous years. Diagnostic plots of the model showed that the model fitted well. The true positive rate was 73%, the true negative rate was 85%, and the 0/1-loss was 15.3%. The predictor effect plots of the important covariates are shown in Figs. 4 and 5.

Night in year was an important predictor for bat presence at sea (Fig. 4A). In autumn, the first Nathusius’ pipistrelle was recorded on 17 August 2020 and the last on 5 December 2019, with the majority passing through in September and October. There is a peak from early to late September, followed by a decrease till mid-November, interrupted by a possible smaller second peak at the end of October. We found an effect of the lunar phase on Nathusius’ pipistrelle occurrence (Fig. 4B). Bat presence was reduced between the full moon and last quarter and increased just before the new moon.

Both tailwind and crosswind (Fig. 4C, D) are important covariates. The highest occurrence of Nathusius’pipistrelle takes place during tailwind conditions (from ENE) of approximately 2 m/s. A further increase in windspeed does not coincide with higher occurrence but even leads to an apparent decrease. In headwind conditions, the occurrence is still positive. The occurrence during crosswind peaks at a wind speed of 1 m/s from the SSE. Increasing crosswinds (from either SSE or NNW) decreased bat presence.

The occurrence of Nathusius’ pipistrelle showed a decrease in 2019 and 2020, a significant difference (*p* < 0.0001) was found between 2020 and the previous years (Fig. 4E).

The predictor effect plot of the spatial smoother for longitude and latitude (Fig. 5) showed that areas in the west had a higher probability of bat presence, while areas in the south, east and north had a lower probability.

## Discussion

Our study provides important information on the spatiotemporal occurrence of Nathusius’ pipistrelle during autumn migration over the southern North Sea as well as on the environmental factors that shape the species’ offshore occurrence.

### Spatiotemporal offshore occurrence

We recorded bats at all monitoring locations, mainly during the night, but also during daylight hours. Frequently, multiple occurrences were recorded during one night at a particular location. These occurrences can be used as a proxy for the presence of bats, since acoustic monitoring does not allow to distinguish between single or multiple individuals (Voigt et al., 2021; Walters et al., 2013). Therefore, it is neither possible to distinguish between single or multiple individuals simultaneously present, nor between a single individual spending a prolonged period at the monitoring location and multiple individuals recorded sequentially.

Offshore occurrence of Nathusius’ pipistrelle during the night differed between our monitoring locations. At locations relatively close to the coast (25–30 km from shore), most individuals were detected between 3 and 6 h after sunset. Given the maximum range speed of 24.8–27.0 km/h which is used during migration (Troxell et al., 2019), we expected the first bats to arrive at the locations closest to shore within 1–2 h after sunset if they start migrating offshore directly after departure from their roost sites in the coastal zone. The delayed arrival at the offshore locations can be caused by bats departing from roosting sites further inland or by bats from coastal sites foraging for a prolonged period above land prior to their North Sea crossing.

At locations further away (> 60 km from shore), however, bats were recorded throughout the night. Frequent observations within an hour after sunset indicate that the recorded animals roosted at the monitoring location or in its vicinity. Diurnal stopovers at sea are also reflected in the results of our statistical analysis, which show an offshore distribution of Nathusius’ pipistrelle with increased bat presence further offshore off the Noord-Holland coast. The increase from east to west can be explained by individuals detected during consecutive nights as they interrupt their migratory flight to roost at sea during daylight hours, thus resulting in an overall higher occurrence further away from the coast.

This remarkable pattern is only present off the Noord Holland coast and seems to be absent further north and further south in our study area. Spatial differences in the number of individuals migrating over the southern North Sea may explain this. Bats prefer to migrate over land and avoid major ecological barriers such as large water bodies as much as possible (Ahlén et al., 2009). This habitat preference can lead to a concentration of migrating bats along the coast (Ijäs et al., 2017; Pētersons, 2004; Šuba et al., 2012), similar to migratory birds (Alerstam, 1993; Berthold, 1990). In the Netherlands, many Nathusius pipistrelles are observed during migration along the Afsluitdijk (Lagerveld et al., 2017), a 32 km dam connecting the provinces of Noord-Holland and Friesland, which are separated by the large freshwater lake IJsselmeer (Fig. 1). Continuing from the Afsluitdijk, bats encounter the Dutch North Sea coast, where they either continue offshore or change direction and follow the coast in an SSW direction. It seems plausible that more bats cross the North Sea in the wake of the Afsluitdijk, resulting in a higher probability of bat presence off the coast of Noord-Holland than in other coastal areas. In addition, the orientation of the North Sea coastline changes from a north-northeast (NNE)–south-southwest (SSW) orientation in the northern part of Noord Holland to a more northeast (NE)–southwest (SW) orientation in the southern part of the Netherlands and ENE–WSW in Belgium. The latter orientation corresponds to the likely main migration direction. Therefore, further south, bats could be more inclined to follow the coastline, resulting in a lower probability of offshore presence in the southern part of our study area.

Our model results showed a peak from early to late September and a subsequent decrease, interrupted by a possible smaller second peak at the end of October. This pattern reflects the general timing of the species’ migration from the breeding areas to the wintering areas (Lagerveld et al., 2014; Pētersons, 2004; Rydell et al., 2014) and is consistent with the results from our earlier study on Nathusius’ pipistrelle at three offshore wind farms within 25 km off the Dutch mainland coast from 2012–2016, showing a peak early September and a subsequent decline till the end of the studied period around mid-October (Lagerveld et al., 2021). The apparent increase at the end of October in our present study may point to gender and/or age-specific differences in timing and extent of migration, which has been found for several other bat species (e.g. Jonasson & Guglielmo, 2016; Petit et al., 2001). In Europe, ring recoveries and isotope analysis revealed female-biased migration for common noctules. Females migrate longer distances than males, whereas males are more sedentary or local migrants (Lehnert et al., 2018; Petit & Mayer, 2000). Nathusius’ pipistrelles’ migration strategy shows similarity to common noctules’ strategy. The first peak coincides with the timing of departure from the breeding areas and migration towards the wintering areas (e.g. Hüppop & Hill, 2016; Šuba et al., 2012), presumably mainly consisting of females and juveniles (Pētersons, 2004). Records later in the season probably consist predominantly of adult males (Jarzembowski, 2003; Troxell et al., 2019). During the autumn migration season, adult males stay along the migration route and try to attract passing females in order to mate with them (Jahelkova & Horacek, 2011). After the mating season, some males migrate to wintering areas, while others stay to winter in the same area (Pētersons, 2004; Sachanowicz et al., 2019).

Finally, we found a significant difference in the occurrence of Nathusius’ pipistrelle between 2020 and the previous years. Although 2017–2020 is a short period to draw firm conclusions, a decline cannot be ruled out. Unfortunately, there is a lack of data on systematically monitored Nathusius’ pipistrelle to verify or falsify a decline.

### Effect of environmental factors on the offshore occurrence

Of the suite of environmental variables, we used to predict the offshore occurrence of Nathusius’ pipistrelle, tailwind, crosswind and lunar phase proved to be important predictors. Precipitation and a change in atmospheric pressure had a minor influence on the offshore presence, whereas cloud cover and atmospheric pressure were found to be not important.

Acoustic monitoring in our earlier study from 2012 to 2016 at three offshore wind farms close to the Dutch coast revealed that wind direction had a marked influence on bat presence at sea (Lagerveld et al., 2021). The offshore occurrence in autumn peaked with a wind direction from ENE. We used this wind direction to calculate the tailwind and crosswind components of the wind vector. The current study found the tailwind component to predict the highest occurrence of Nathusius’ pipistrelle with a wind speed of 2 m/s from the ENE. A further increase in wind speed does not result in an increased bat presence but even leads to an apparent decrease. Furthermore, we found that bat occurrence is still positive in low to moderate headwind conditions and with low to moderate crosswinds, in particular during crosswinds from land. In other words, bats also occur at sea with low to moderate headwinds or crosswinds, but they prefer to do so with supportive tailwind. Tailwind was also an important factor for bat occurrence off the Belgian coast (Brabant et al., 2021) and off the eastern coast of the United States (Pettit & O’Keefe, 2017). Low to moderate wind speeds are thought to be the preferred conditions for bats to migrate (Ahlén et al., 2009; Brabant et al., 2021; Hüppop & Hill, 2016; Lagerveld et al., 2014, 2021).

The observed decrease in bat presence with higher wind speeds from the ENE in our study may be an artefact: migrating bats, theoretically, adapt their flight altitude to optimise the use of tailwinds (Hedenström, 2009). If they fly higher to take advantage of the stronger wind speeds at higher altitudes, they can fly above the detection range of our acoustic equipment (> 50 m above sea level), resulting in the under-recording of acoustic activity during these conditions. Bat migration at relatively high altitudes over sea during strong tailwind conditions has been shown by Hatch et al. (2013), who photographed multiple bats at heights of over 200 m off the US east coast with tailwinds up to 10 m/s. It is, therefore, important to gather information on the flight height during migration in relation to wind, in order to verify the observed relationship between migration intensity and wind support.

We found that increasing crosswinds (from either SSE or NNW) decreased bat presence, indicating that moderate or high crosswinds are avoided. Bat occurrence peaks at a wind speed of 1 m/s from the SSE, indicating that crosswinds from land are preferred over crosswinds from sea. It may, therefore, also be possible that wind drift caused by crosswinds from land plays a role in the offshore occurrence of bats, as suggested by Hüppop and Hill (2016) who recorded bats in the German Bight mainly during southerly winds from the mainland.

Rain increases the energy expenditure during flight significantly (Voigt et al., 2011). We found a minor (negative) influence of precipitation on the offshore presence (*p* = 0.09), in accordance to previous studies which showed that bats avoid the rain while migrating over the North Sea (Brabant et al., 2021; Hüppop & Hill, 2016; Lagerveld et al., 2014). In the German Bight, however, most bats are recorded in rainy and overcast conditions, likely as a result of a change of weather during their migratory flight over sea (Hüppop & Hill, 2016). Increased bat presence during overcast conditions was also noted off the western coast of the United States (Cryan & Brown, 2007). Therefore, offshore bat activity may not only be the result of favourable conditions to cross over sea, but may also be the result of stopovers due to deteriorating weather after departure. The (weak) positive relationship between bat occurrence and decreasing atmospheric pressure we found indicates the latter also occurred during our study.

In addition to the forementioned relationships between environmental variables and the occurrence of Nathusius’ pipistrelle, our study showed a reduced offshore occurrence between the full moon and the third quarter and indicate an increase just before the new moon. A reduced occurrence of migratory bats with higher moon illumination was found off the western coast of the United States (Cryan & Brown, 2007).

Predation risk and insect availability are postulated as important factors shaping activity patterns in response to moon illumination, as insectivorous bats are both predator and prey (Kronfeld-Schor et al., 2013; Lang et al., 2006). Predators of Nathusius’ pipistrelle and other insectivorous bats include several species of owls, diurnal raptors, gulls and crows (Speakman, 1991; Sieradzki & Mikkola, 2020). Although bats constitute only a small proportion of the diet of aerial predators in Europe (Speakman, 1991; Sieradzki & Mikkola, 2020), bats reduce predation risk by their choice of foraging areas (Baxter et al., 2006) and by their timing of roost departures (Lima & O’Keefe, 2013). Predation risk is likely to increase with higher moon illumination, since bats become more visible. However, most insectivorous bats from temperate areas do not seem to avoid moonlit nights (Lima & O’Keefe, 2013). In Europe, so far, only Daubenton’s bat *Myotis daubentonii* was found to reduce its activity significantly during higher phases of the moon (Ciechanowski et al., 2007). This study also found an almost significant negative relationship between Nathusius’ pipistrelles activity and moon illumination (Ciechanowski et al., 2007). Consequently, we cannot exclude the possibility that predation risk may be a factor affecting migratory movements and thus the offshore occurrence of Nathusius’ pipistrelle.

Another important factor for the occurrence of bats, prey availability, can also show a relationship with the lunar phase (Kronfeld-Schor et al., 2013). The diet of Nathusius’ pipistrelle mainly consists of Chironomidae (Diptera) (Beck, 1995), of which some species are known to adjust their emergence to specific phases during the lunar cycle (Danthanarayana, 1976). In autumn, insects from coastal areas may disperse over sea, when this coincides with easterly winds. Insect migration activity may also be associated with the lunar phase (Danthanarayana, 1976). In particular, during late summer/early autumn, many insects migrate over sea (Chapman et al., 2004; Drake & Gatehouse, 1995; Drake & Reynolds, 2012). They are likely attracted to illuminated offshore platforms, thereby facilitating bats to forage in the vicinity of these platforms during their migratory flight over sea (e.g. Ahlén et al., 2009; Šuba et al., 2012). Offshore foraging by migratory bats has been confirmed by our study, as 7.8% of the recordings contained feeding buzzes. Thus, the observed relationship between the lunar phase and offshore bat occurrence may be caused by reduced migratory bat activity during higher phases of the moon to avoid predation, by bats making use of increased insect availability over sea, or by a combination of both factors.

## Conclusions

Our study provides the first comprehensive analysis of the spatiotemporal occurrence of migrating bats over sea. It shows that autumn migration of Nathusius’ pipistrelle over the southern North Sea occurs from mid-August until the end of October. Most bats can be expected off the Noord Holland coast. In particular at locations further away from the coast diurnal stopovers regularly occur, indicating that overseas crossings frequently last longer than one night. Migration peaked during weather conditions with supportive winds from the ENE. Offshore bat presence proved to be affected by the lunar phase which may point to a relationship with predation risk, increased insect availability, or both.

The observed offshore distribution can be used in spatial planning of future offshore wind farms, whereas the temporal occurrence and environmental factors that shape Nathusius’ pipistrelle offshore migration can be used to develop and finetune mitigation measures to reduce the number of bat fatalities at offshore wind farms.

### Supplementary Information

Below is the link to the electronic supplementary material.

Supplementary file1 (PDF 63 KB)

Supplementary file2 (PDF 1272 KB)

Supplementary file3 (PDF 489 KB)

## Data Availability

The data used in this study are available on request from the corresponding author. The data are not publicly available yet, but in due time, they will be made publicly available in the data lab from the Dutch Offshore Wind Ecological Programme (WOZEP).

## Abbreviations

ENE: East-northeast
NE: Northeast
NNE: North-northeast
NNW: North-northwest
OHVS: Offshore high voltage station
SSE: South-southeast
SSW: South-southwest
SW: Southwest
WSW: West-southwest
