# Supplementary material for: Acoustic monitoring reveals spatiotemporal occurrence of Nathusius’ pipistrelle at the southern North Sea during autumn migration
Source: Environ Monit Assess. 2023 Aug 2;195(9):1016. doi: 10.1007/s10661-023-11590-2 (PMC10397122; doi:10.1007/s10661-023-11590-2)
Supplement: Supplementary file 1 — Supplementary file1 (PDF 63 KB) [file 10661_2023_11590_MOESM1_ESM.pdf]

Online resource 1: Details on the monitoring locations

Geographical location of the monitoring locations and orientation and heigth of the microphones.

ID	*Location*	*Longitude*	*Latitude*	*Orientation microphone [degrees]*	*Height above sea level [m]*
A	C-power OHVS	2.99	51.57	60	15
B	Belwind OHVS	2.81	51.69	90	20
C	Europlatform	3.27	51.99	90	15
D	Lichteiland Goeree	3.66	51.92	90	15
E	Dana P11-B	3.34	52.35	90	25
F	Luchterduinen OHVS	4.17	52.40	90	15
G	Petrogas P9-A (Horizon)	3.74	52.55	45	33
H	PAWP OHVS	4.23	52.58	90	15
I	Wintershall P6-A	3.75	52.75	110	23
J	Petrogas Q1-A (Helder)	4.09	52.92	200	25
K	Wintershall K13-A	3.22	53.05	130	25
L	Neptune K12-BP	3.89	53.34	135	20
M	Neptune L10A-AC	4.20	53.40	90	17
