# Supplementary material for: Acoustic monitoring reveals spatiotemporal occurrence of Nathusius’ pipistrelle at the southern North Sea during autumn migration
Source: Environ Monit Assess. 2023 Aug 2;195(9):1016. doi: 10.1007/s10661-023-11590-2 (PMC10397122; doi:10.1007/s10661-023-11590-2)
Supplement: Supplementary file 2 — Supplementary file2 (PDF 1272 KB) [file 10661_2023_11590_MOESM2_ESM.pdf]

Online resource 2: Acoustic bat activity of Nathusius' pipistrelle for all monitoring locations

The recorded acoustic bat activity of Nathusius' pipistrelle per monitoring location per year is shown below. In these figures the dots refer to positive minutes with acoustic activity throughout the night (time interval between sunset and sunrise is represented by grey) and throughout the season. The effective monitoring period is indicated by a white background, whereas a blue background indicates no monitoring. The monitoring location and calendar year are indicated in the upper right corner of each figure.

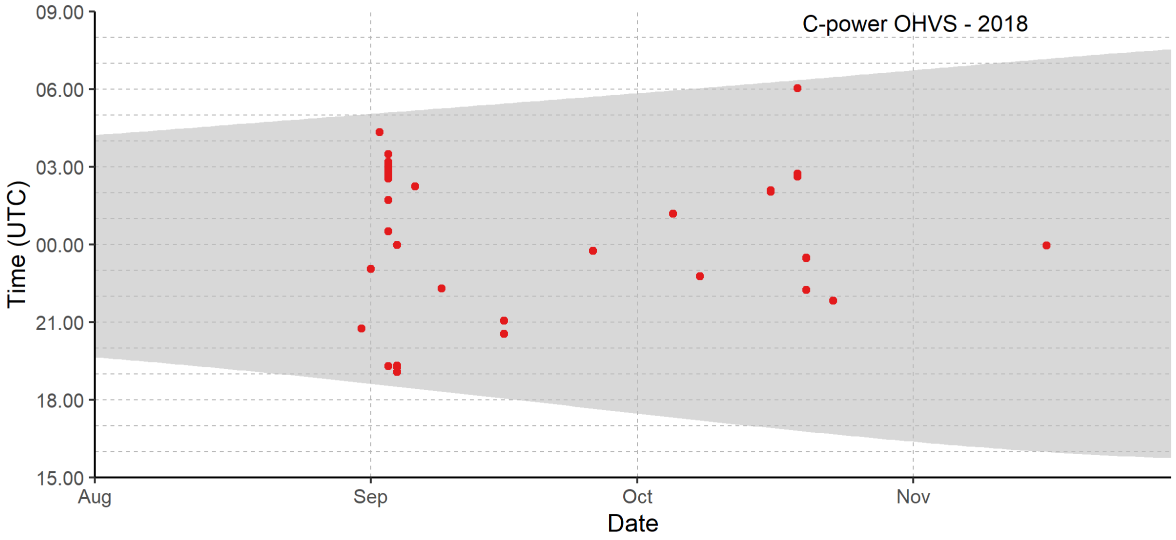

Figure 6.

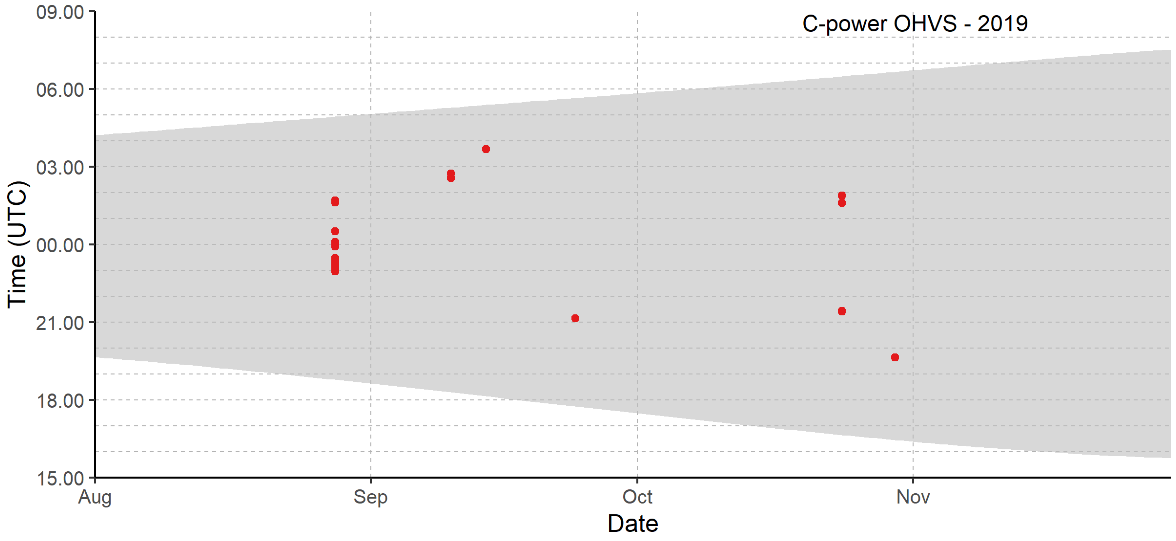

Figure 7.

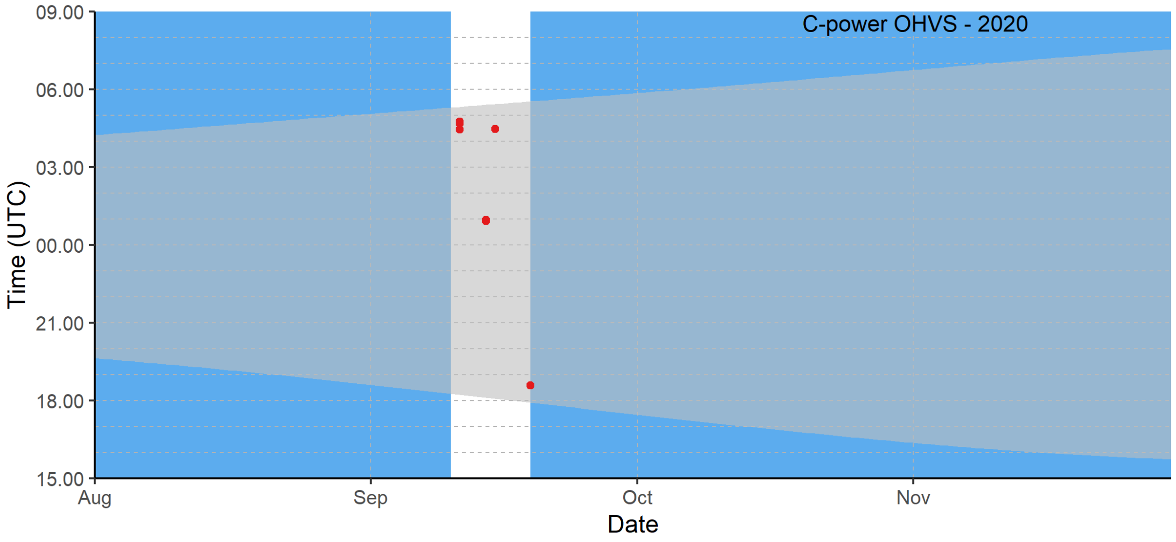

Figure 8.

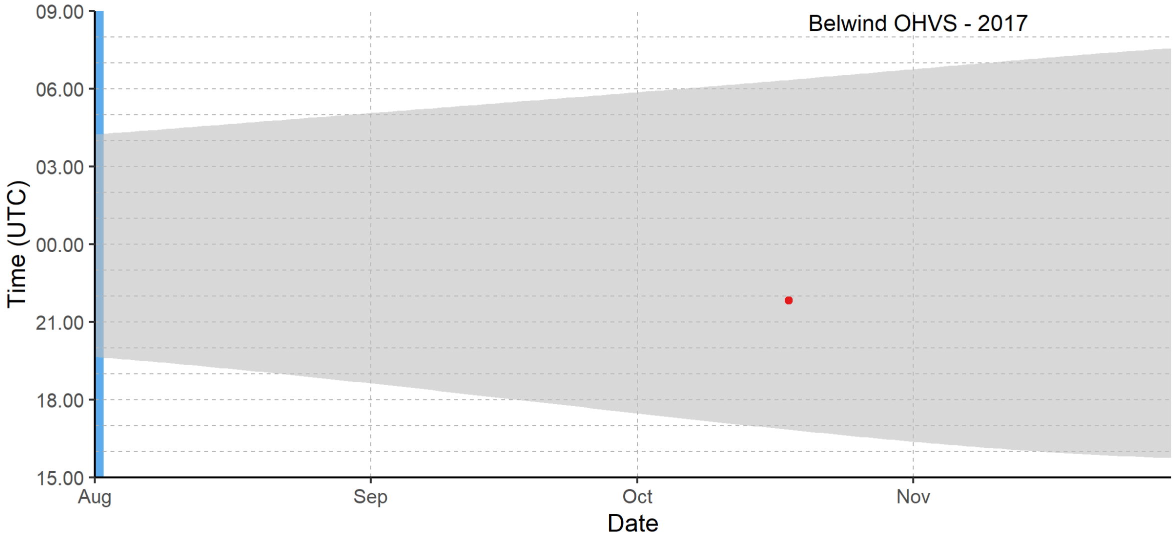

Figure 9.

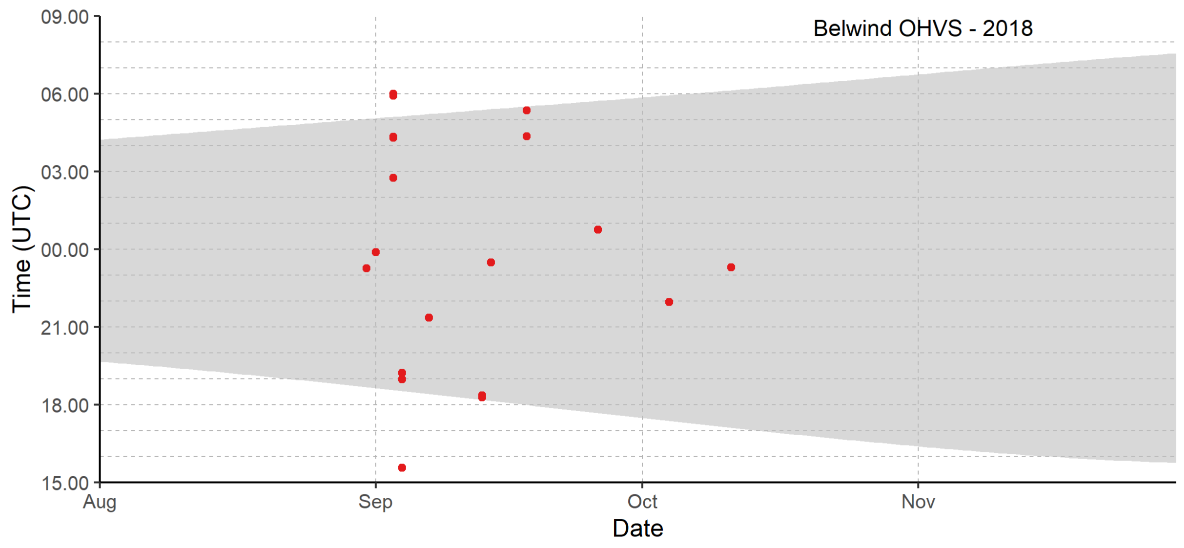

Figure 10.

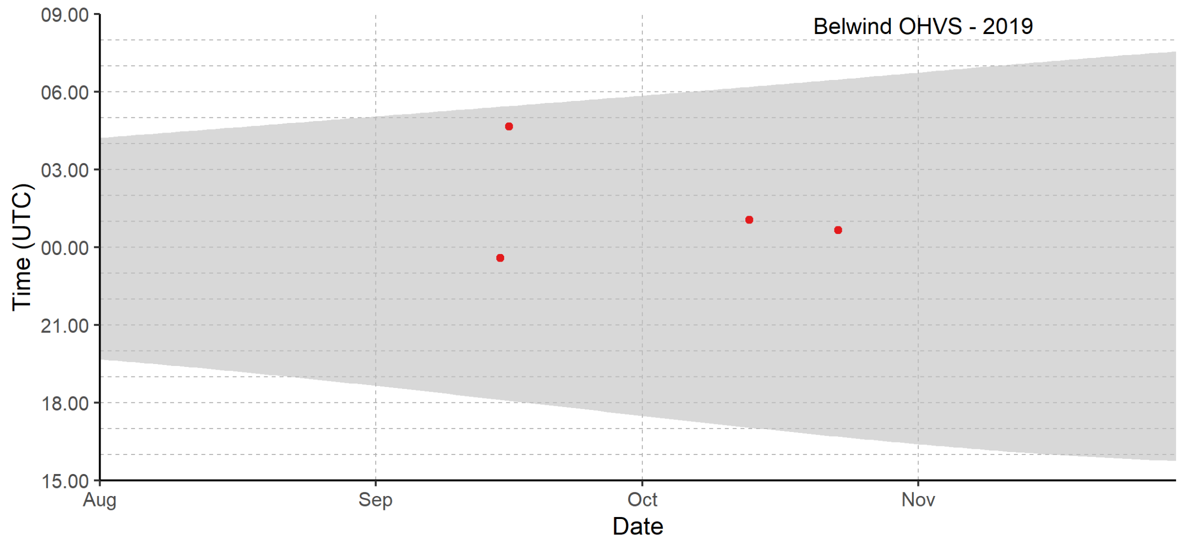

Figure 11.

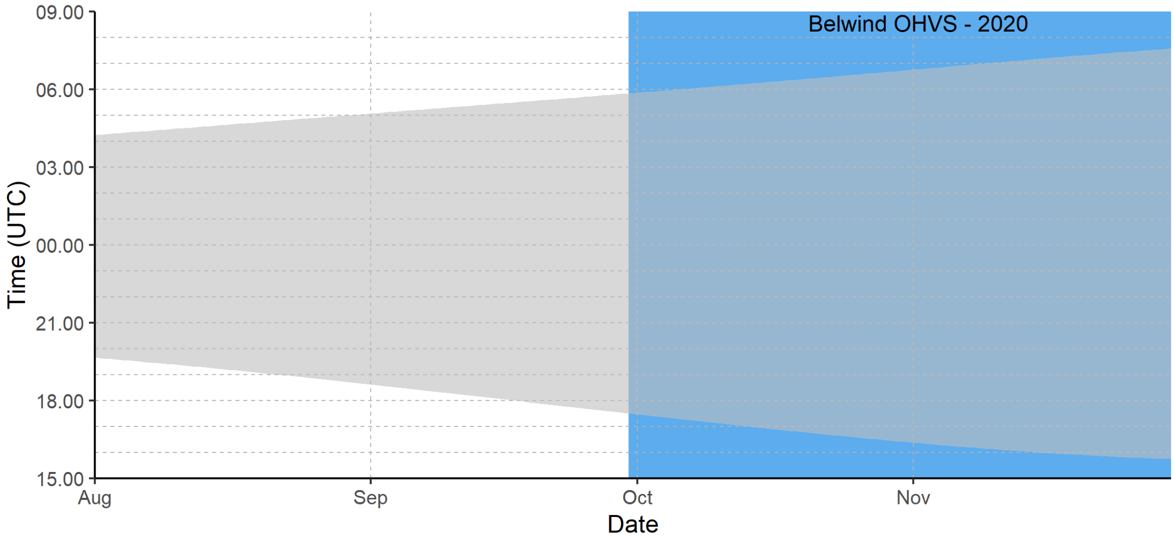

Figure 12.

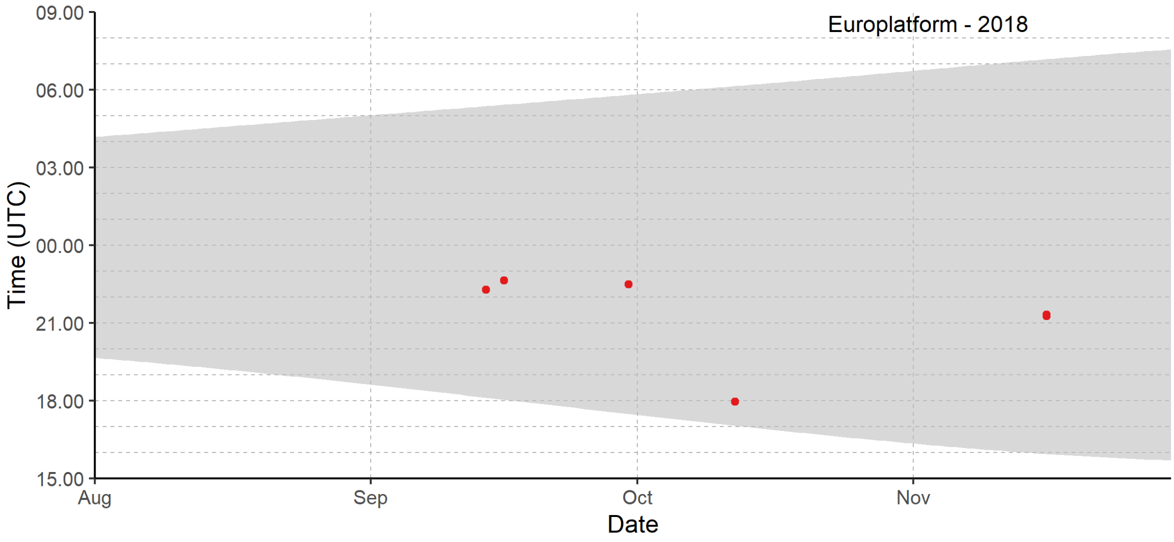

Figure 13.

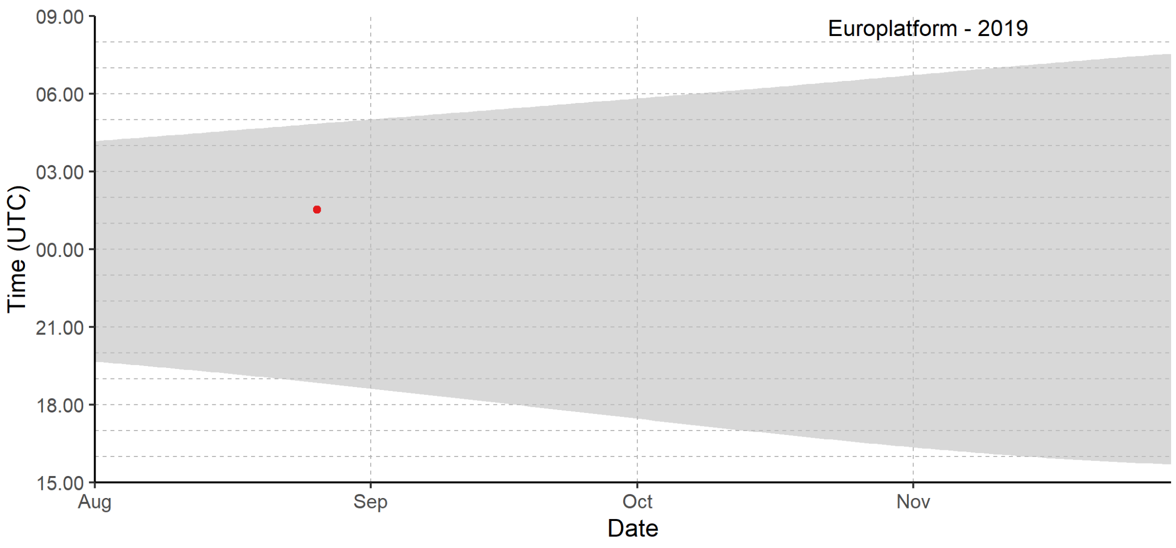

Figure 14.

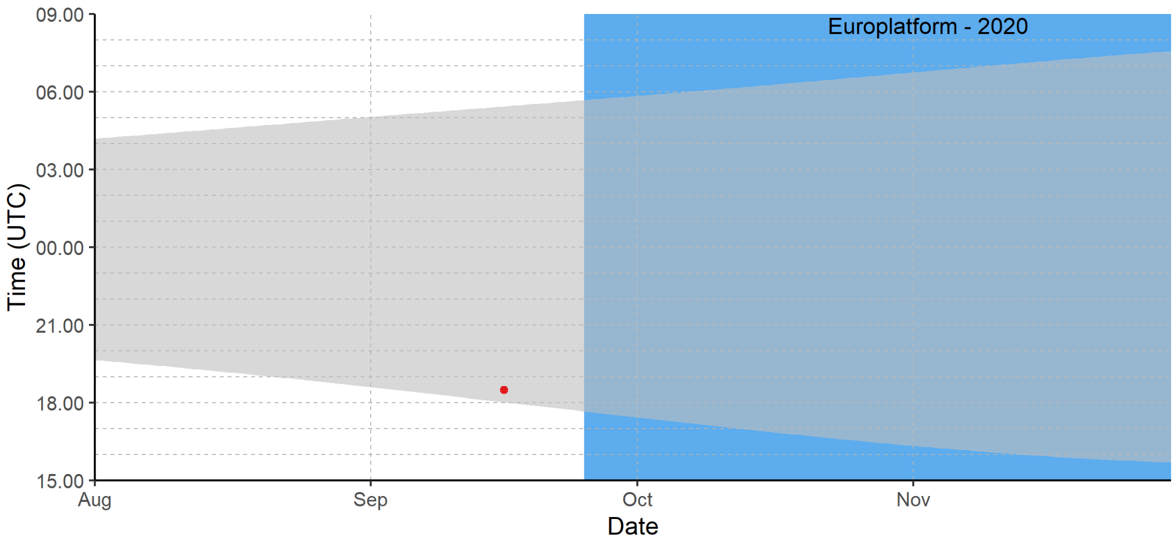

Figure 15.

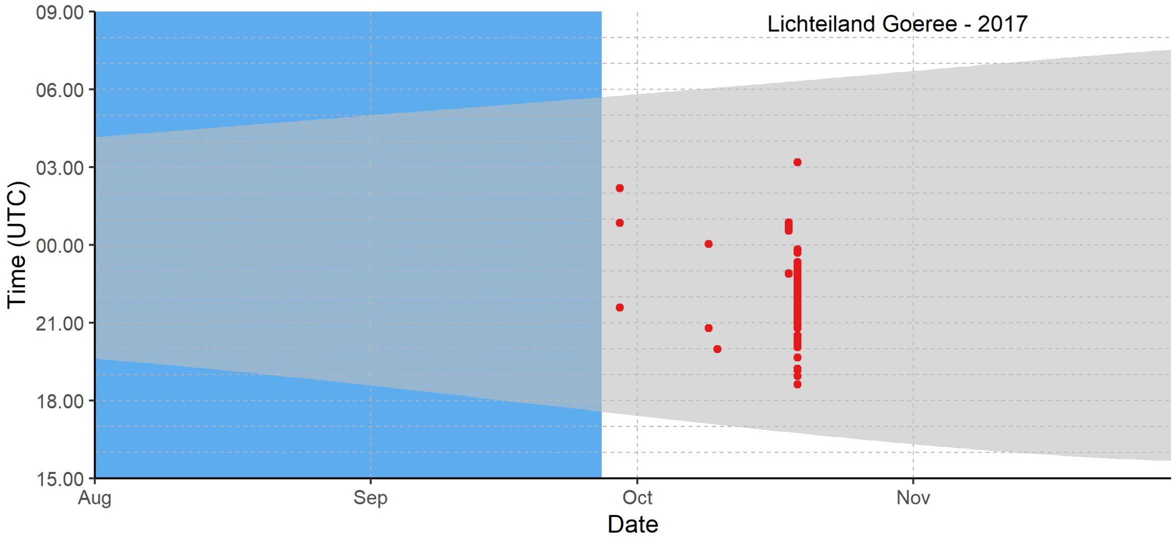

Figure 16.

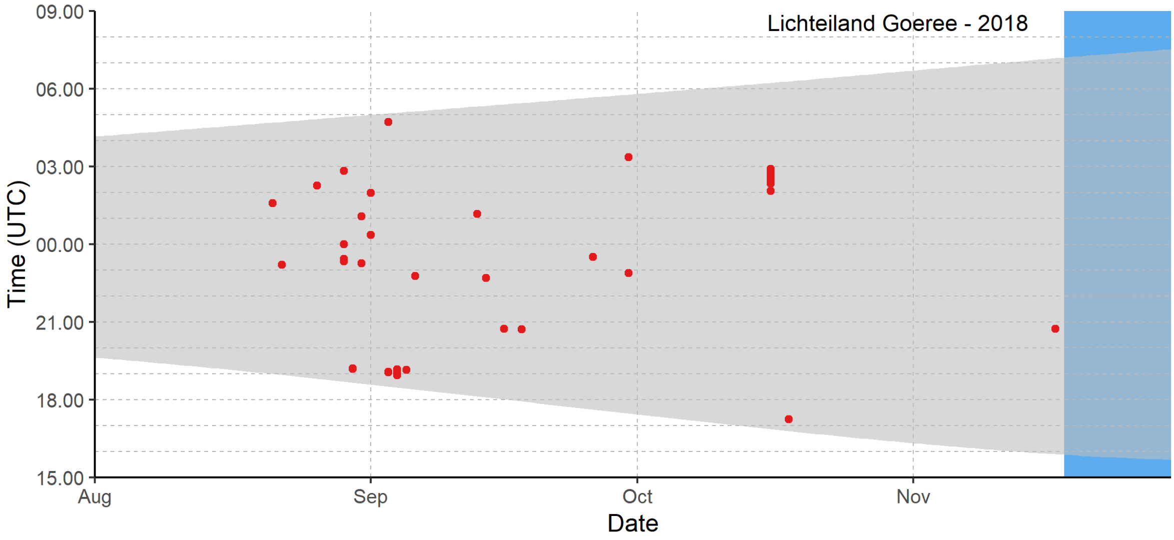

Figure 17.

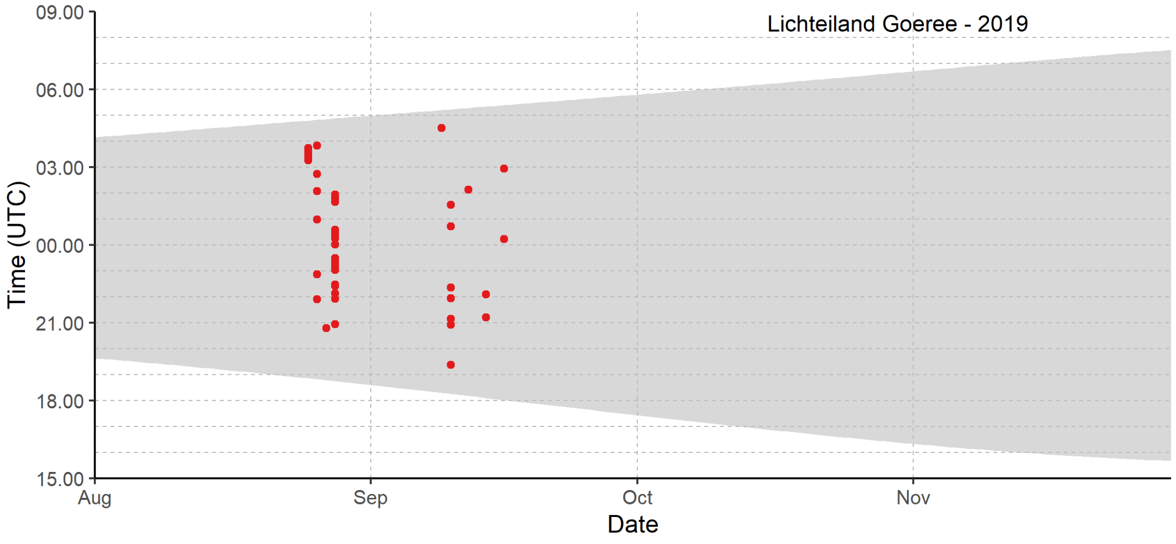

Figure 18.

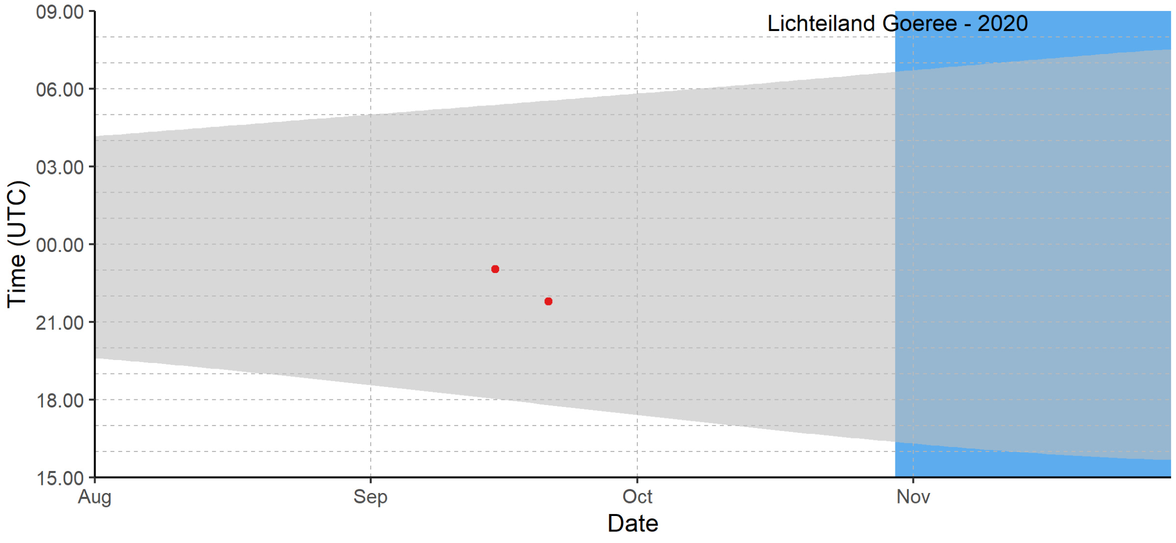

Figure 19.

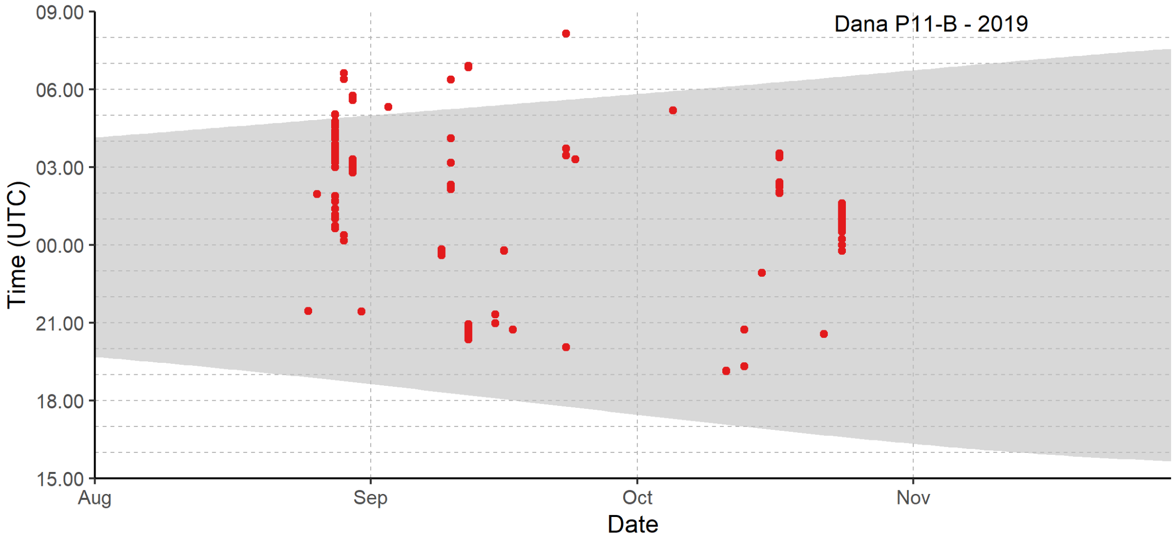

Figure 20.

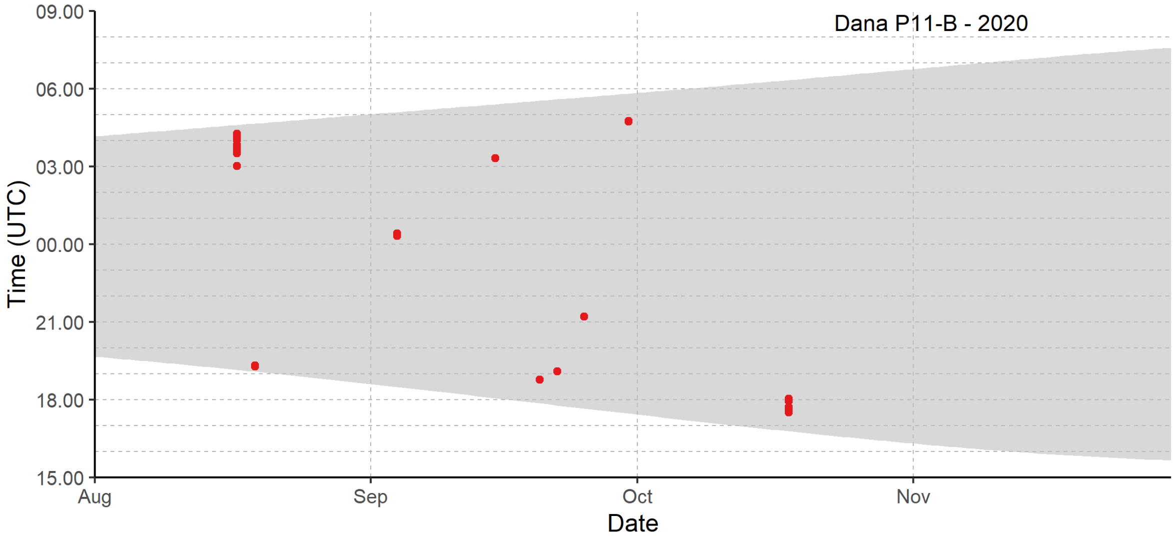

Figure 21.

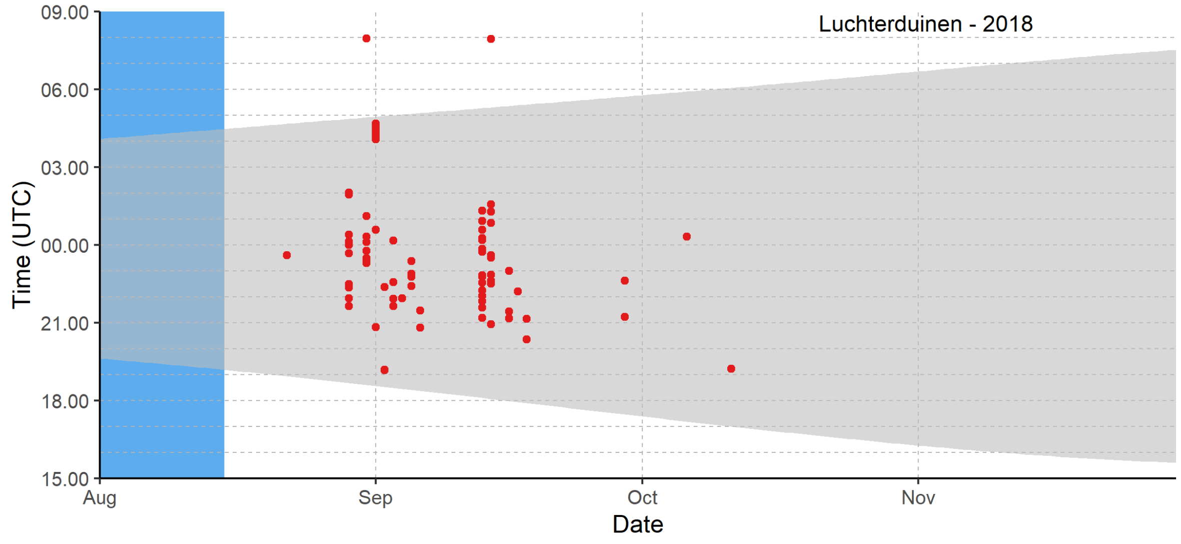

Figure 22.

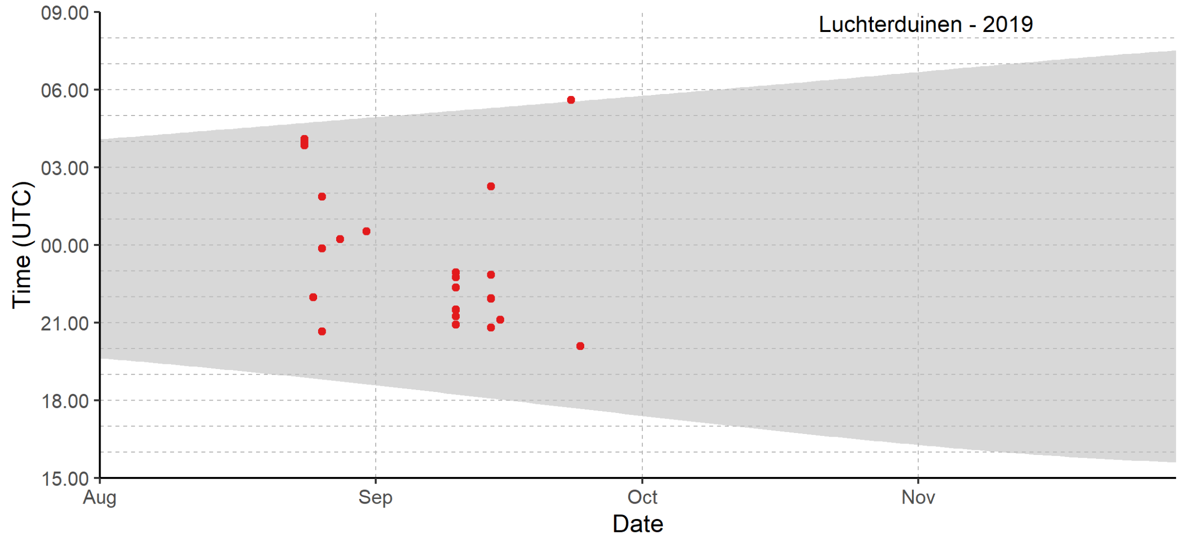

Figure 23.

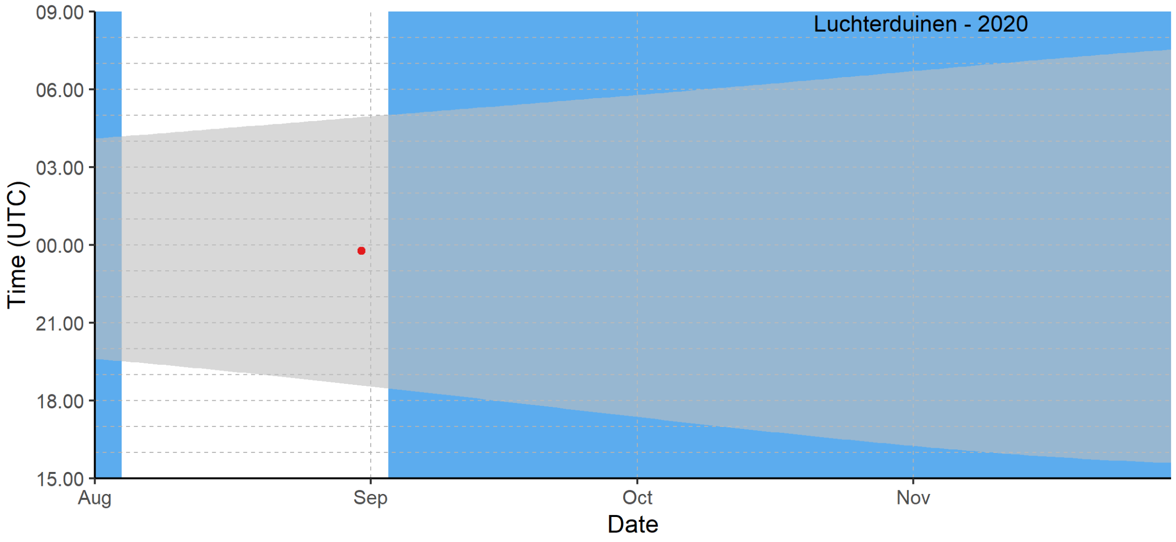

Figure 24.

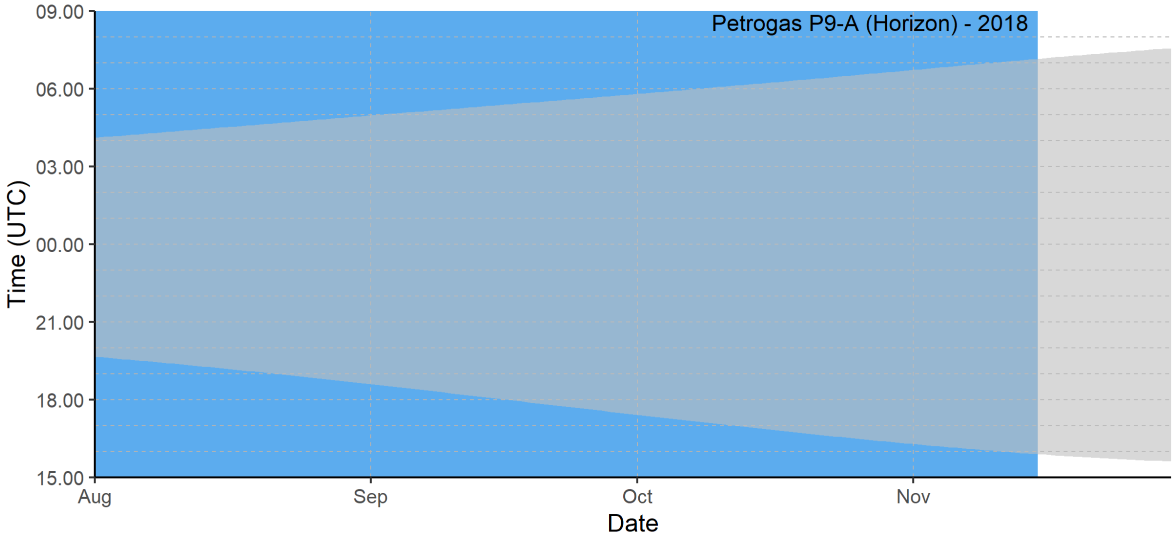

Figure 25.

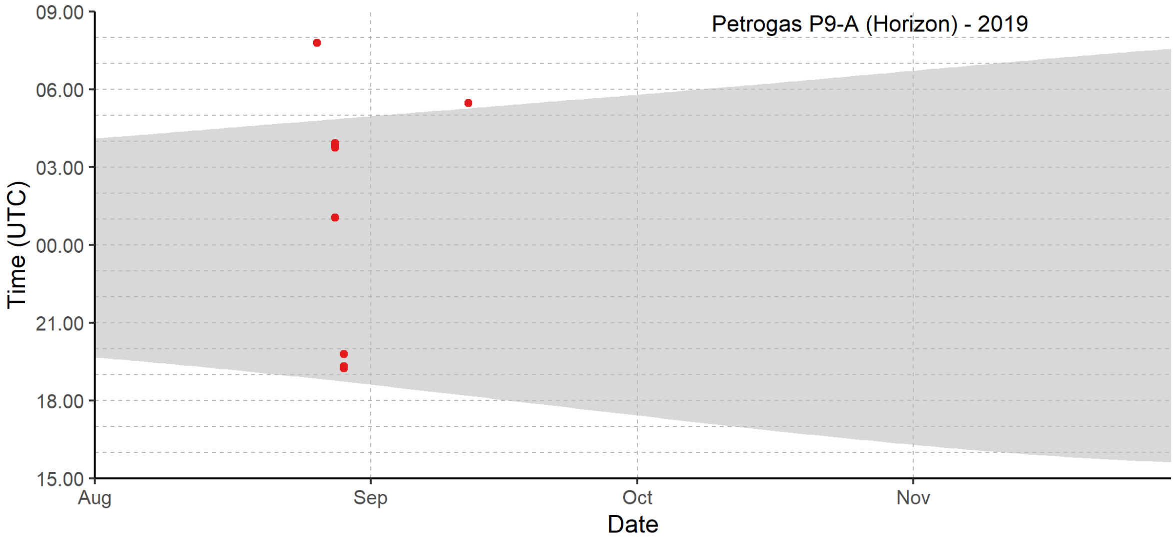

Figure 26.

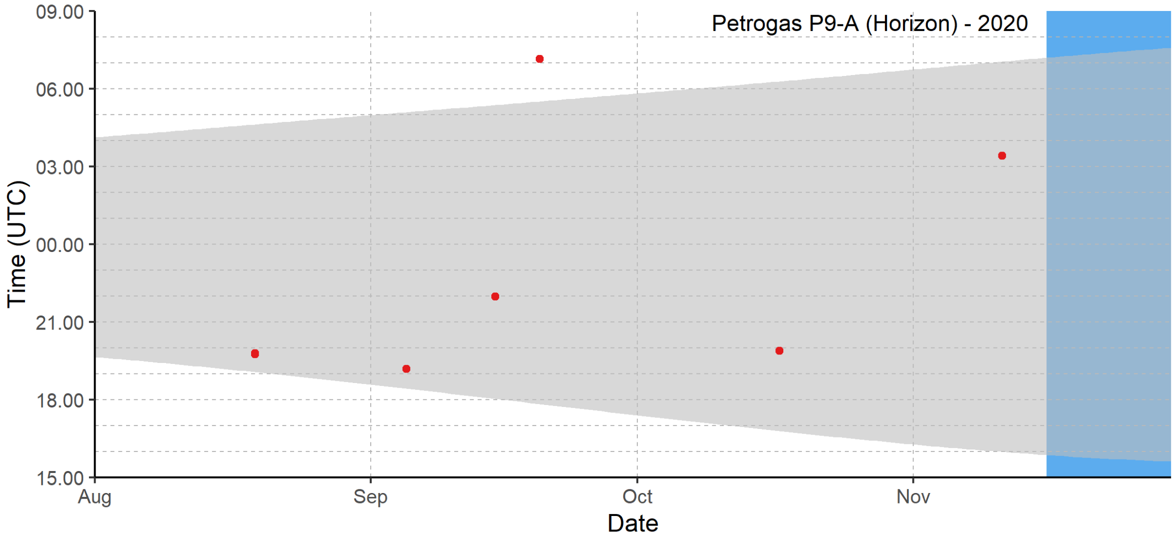

Figure 27.

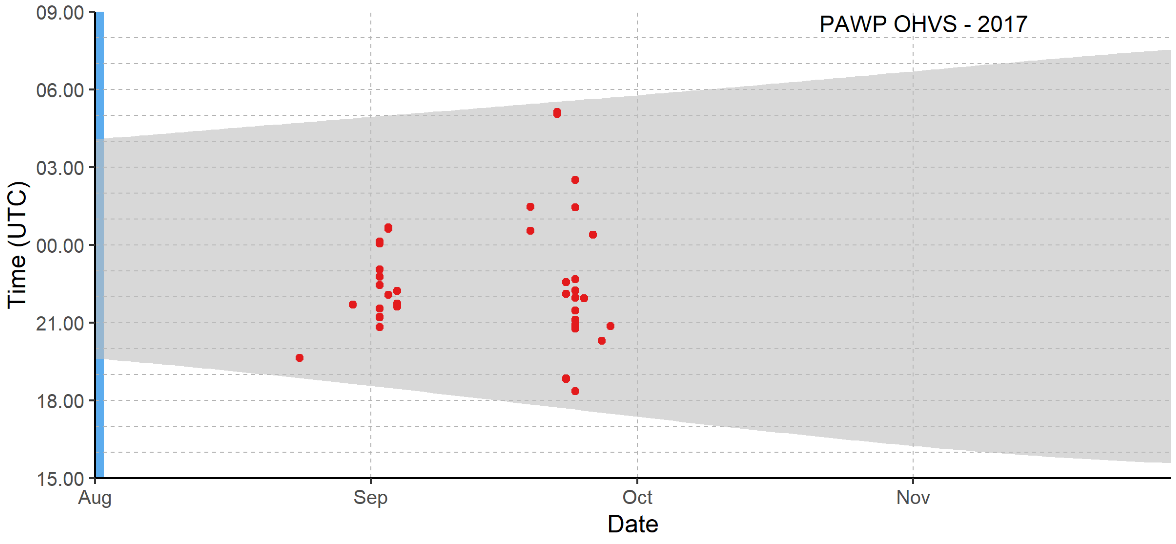

Figure 28.

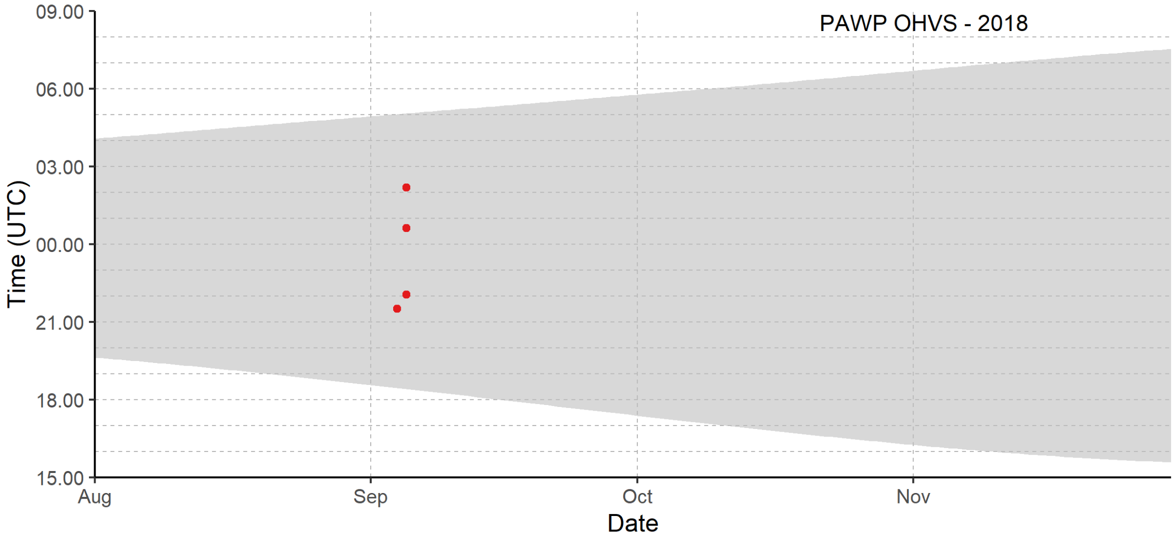

Figure 29.

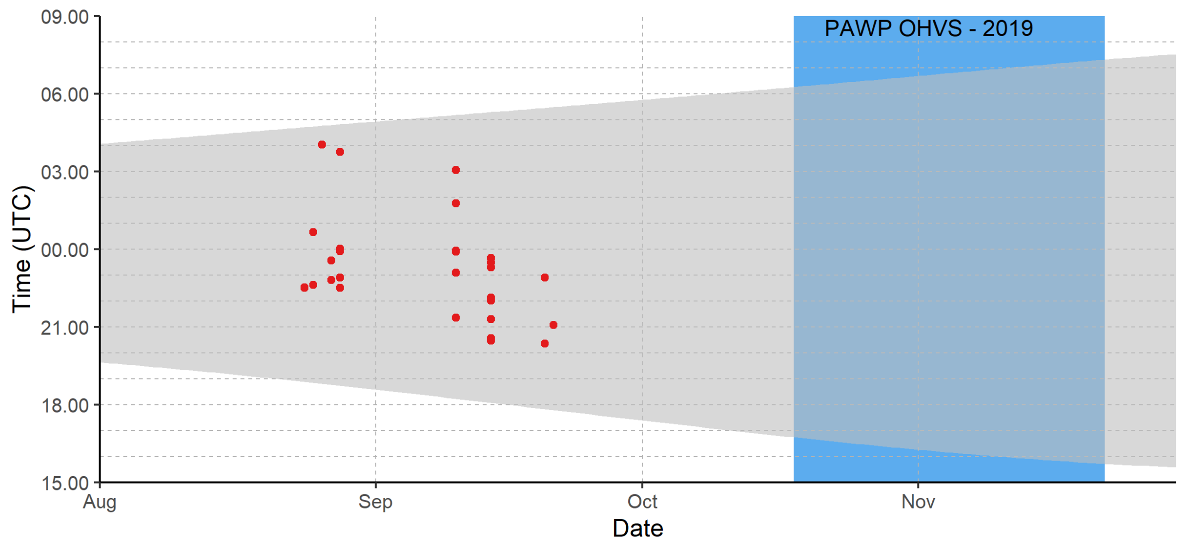

Figure 30. Same as Figure 2 in the main text.

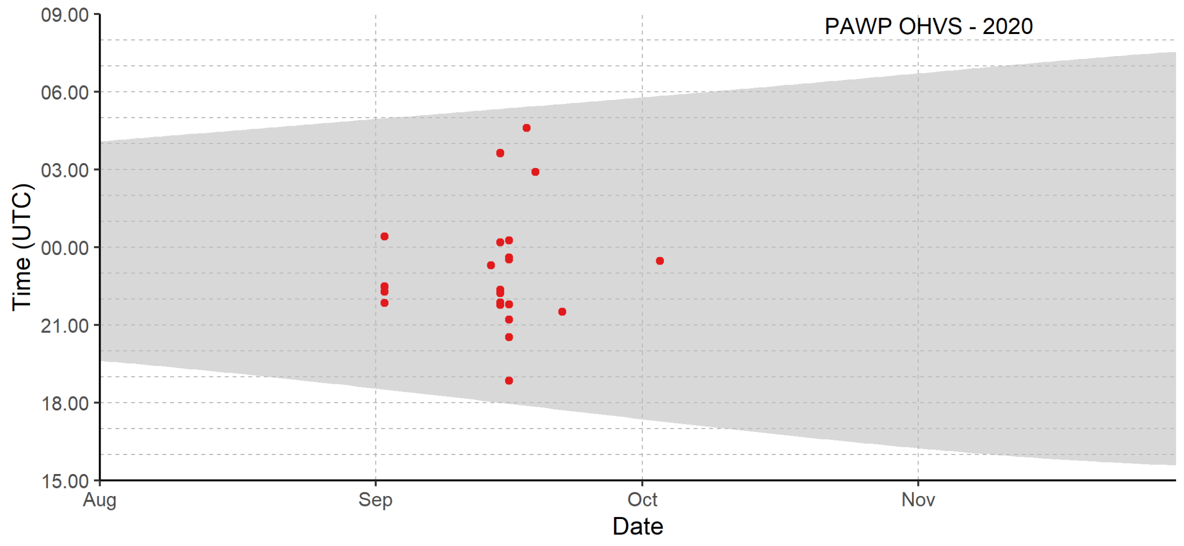

Figure 31.

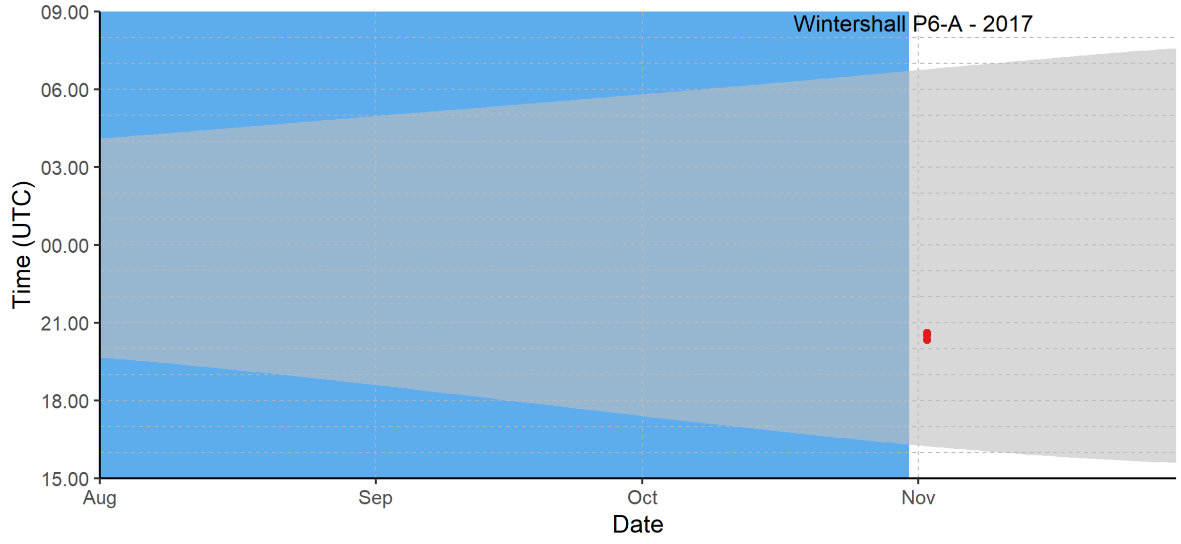

Figure 32.

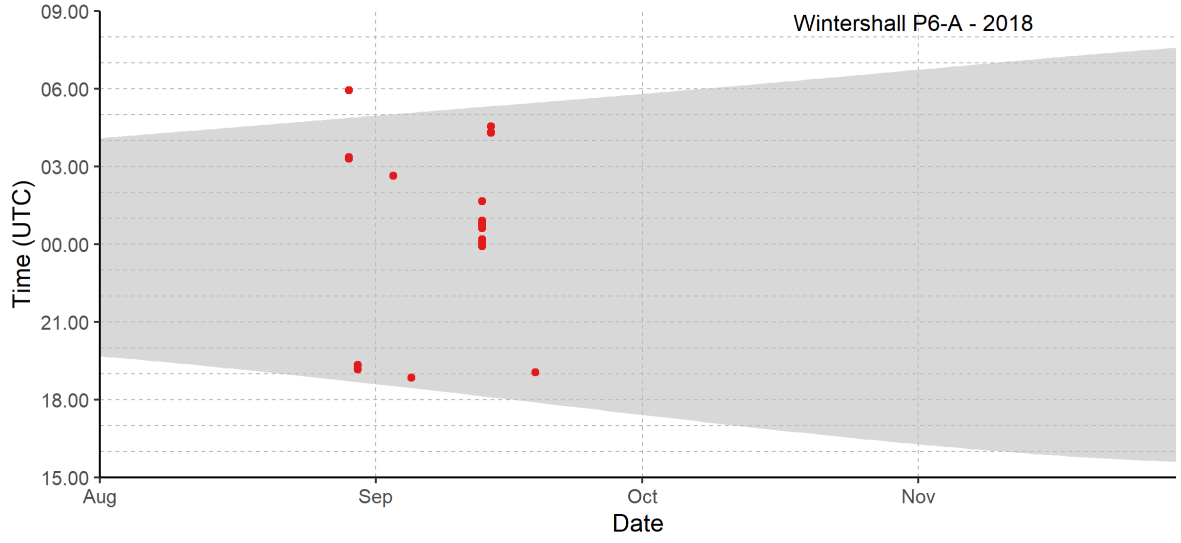

Figure 33.

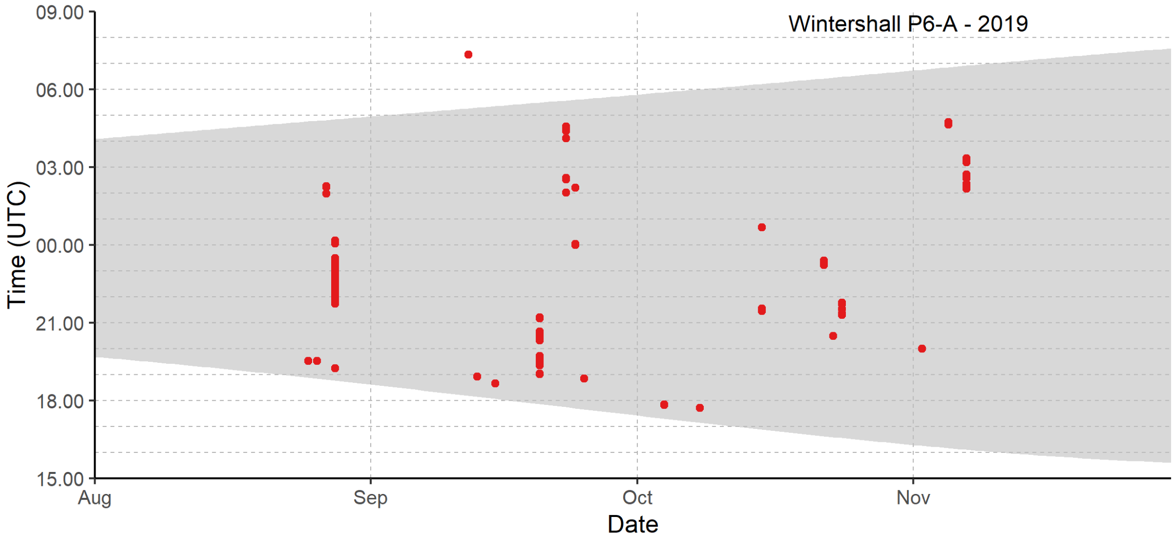

Figure 34. Same as Figure 3 in the main text.

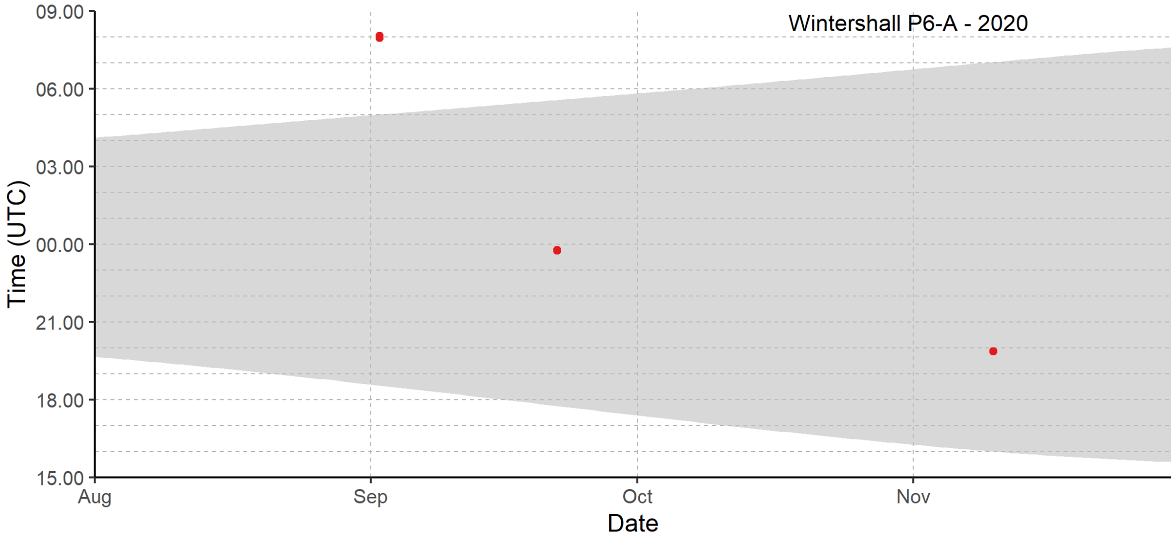

Figure 35.

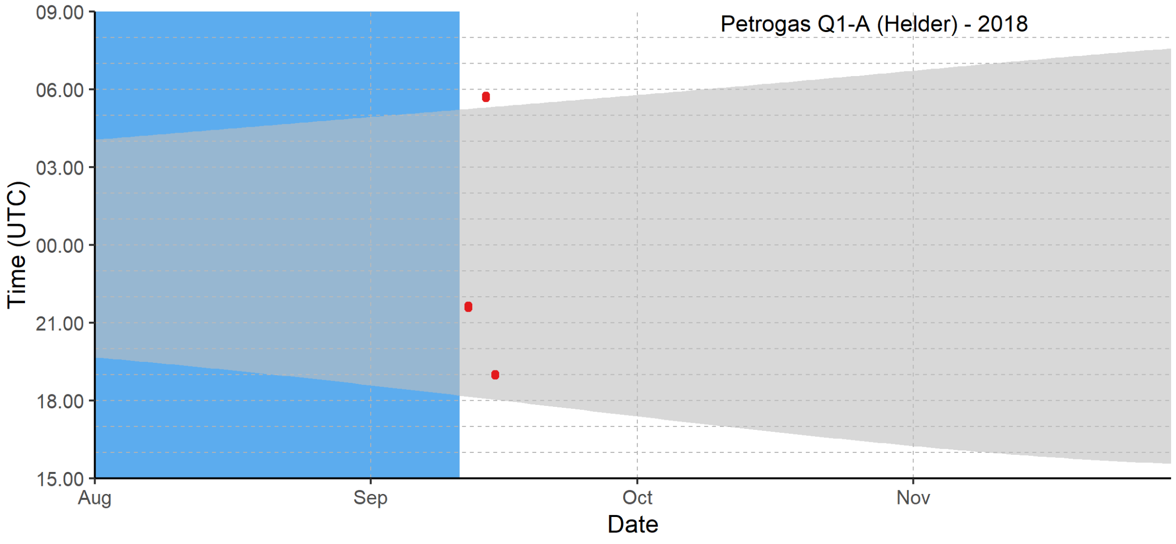

Figure 36.

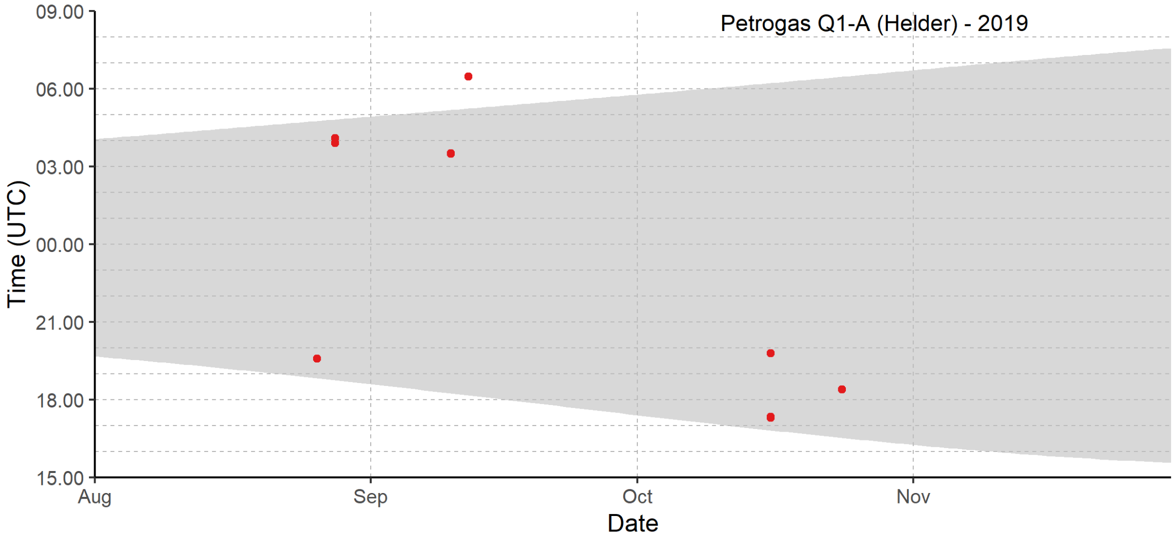

Figure 37.

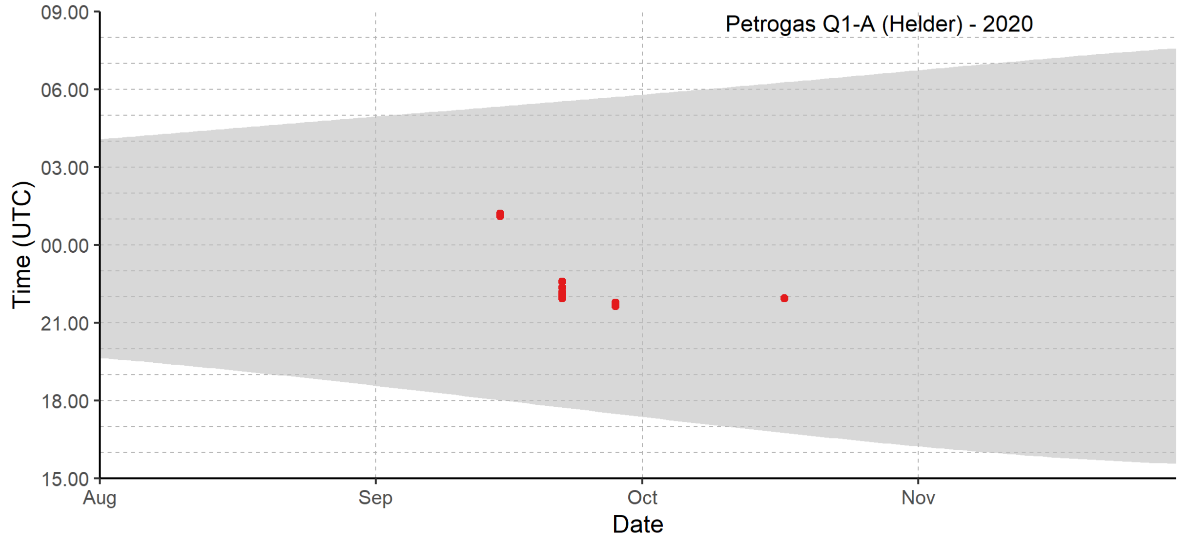

Figure 38.

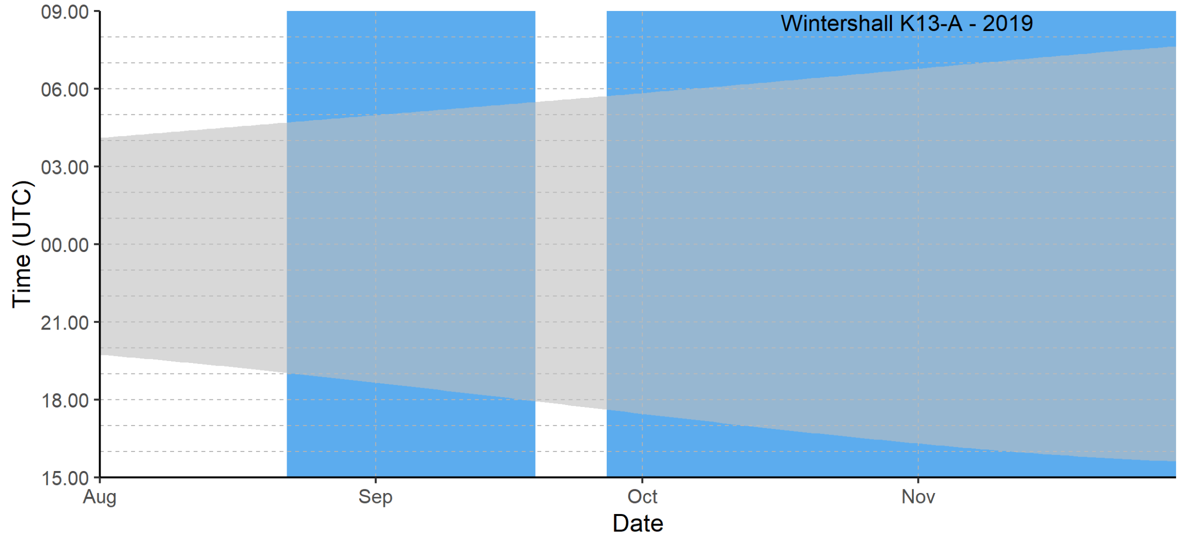

Figure 39.

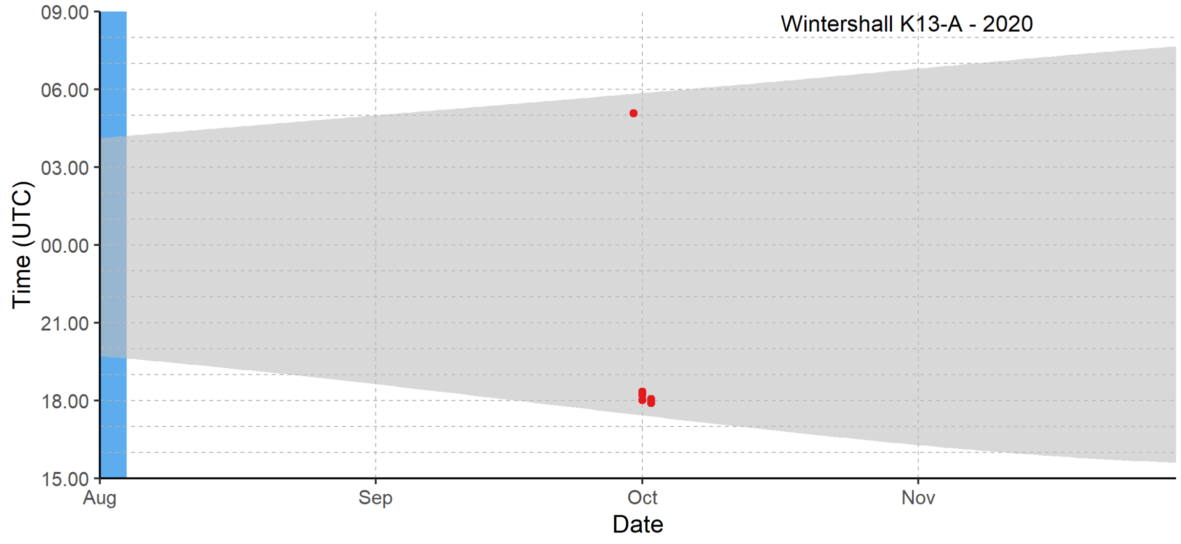

Figure 40.

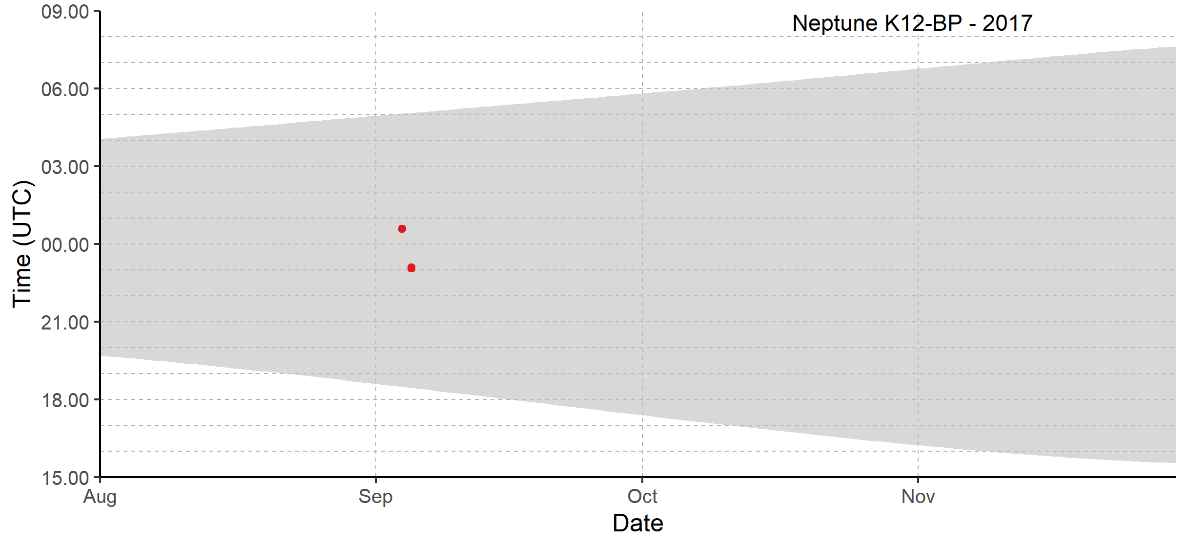

Figure 41.

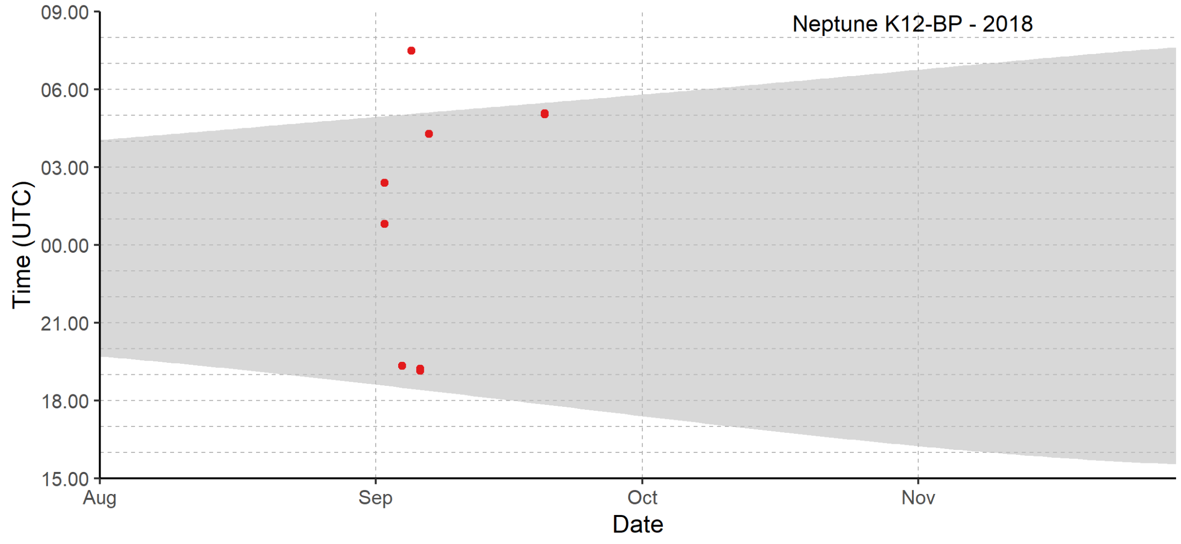

Figure 42.

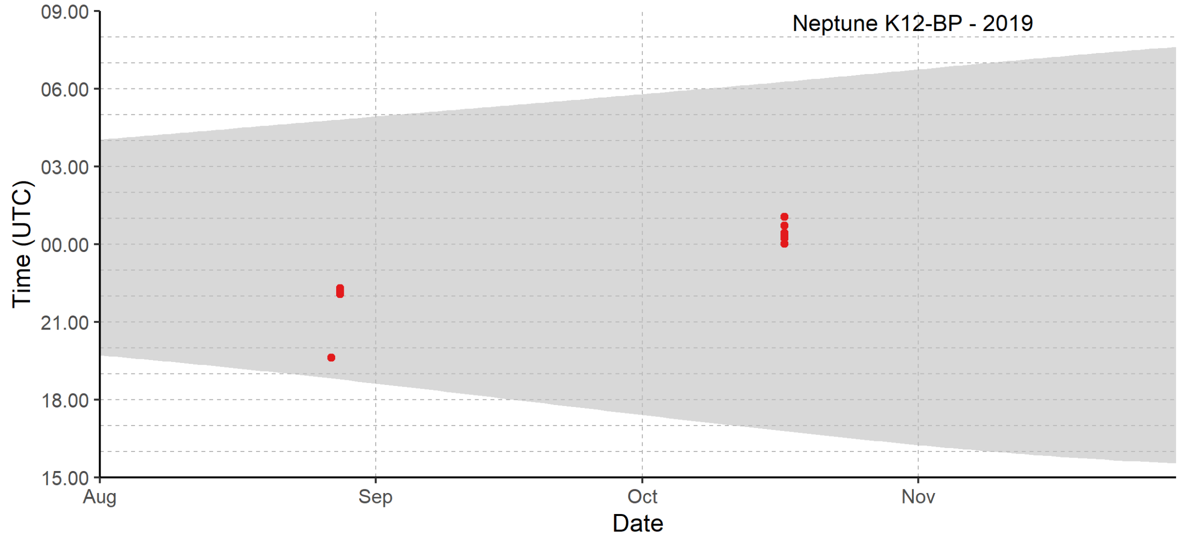

Figure 43.

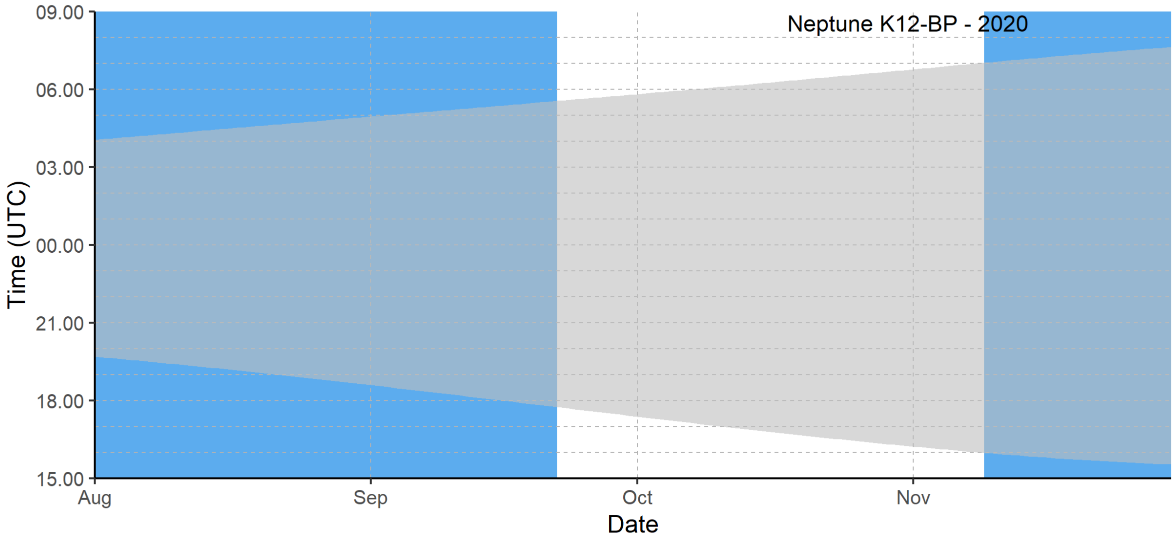

Figure 44.

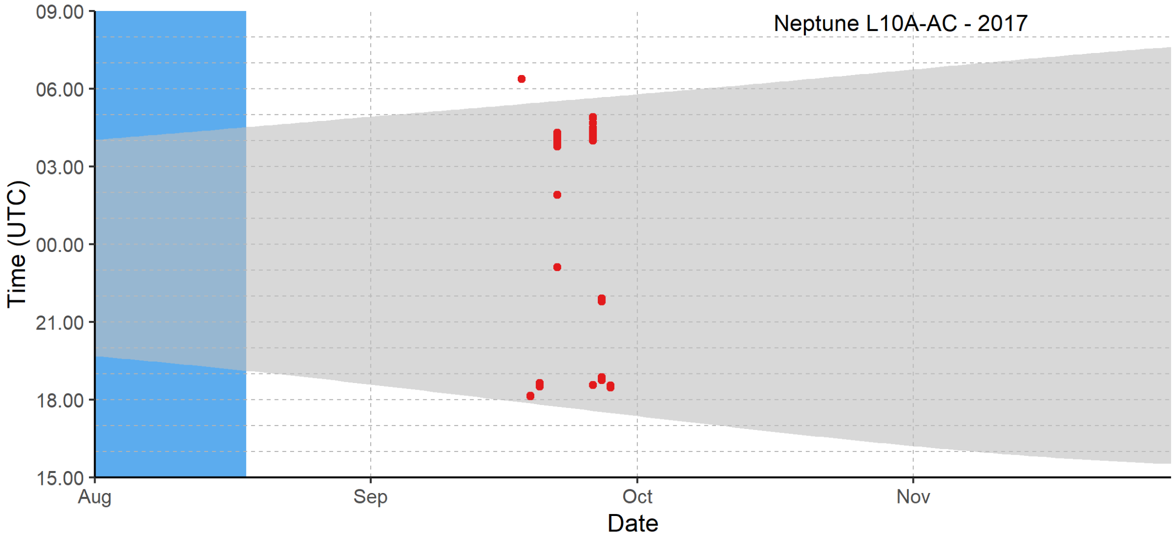

Figure 45.

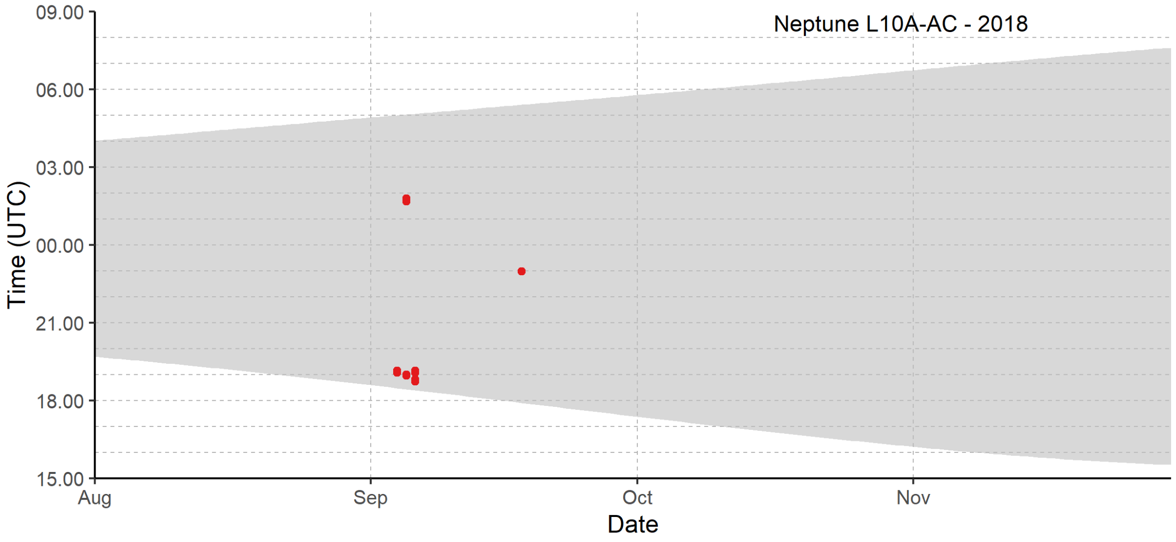

Figure 46.

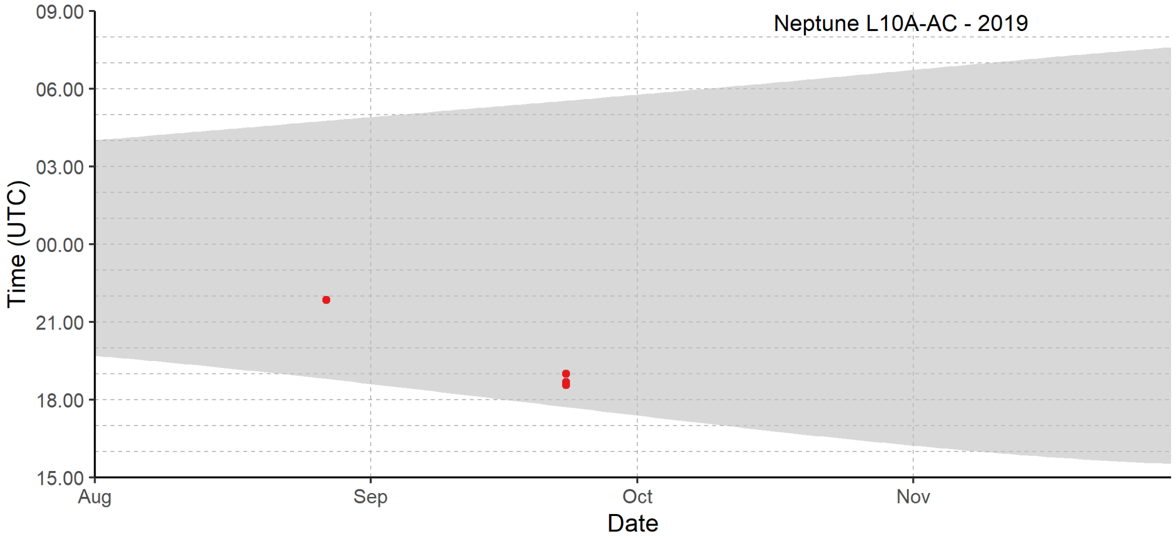

Figure 47.

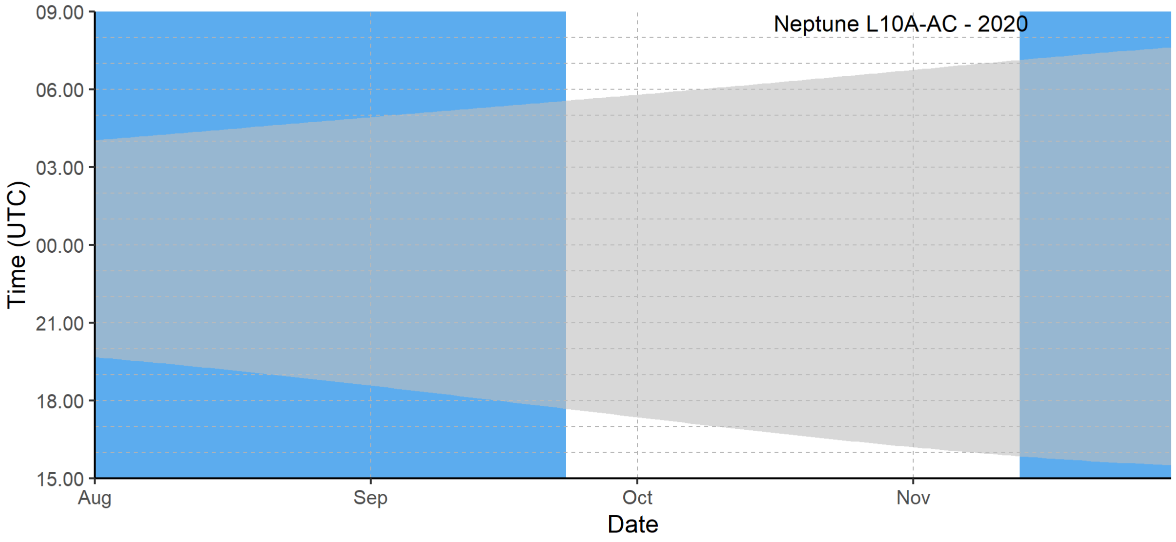
Neptune L10A-AC - 2020
Time (UTC)
09.00
06.00
03.00
00.00
21.00
18.00
15.00
Aug
Sep
Oct
Nov
Date

Figure 48.
