# Supplementary material for: Acoustic monitoring reveals spatiotemporal occurrence of Nathusius’ pipistrelle at the southern North Sea during autumn migration
Source: Environ Monit Assess. 2023 Aug 2;195(9):1016. doi: 10.1007/s10661-023-11590-2 (PMC10397122; doi:10.1007/s10661-023-11590-2)
Supplement: Supplementary file 3 — Supplementary file3 (PDF 489 KB) [file 10661_2023_11590_MOESM3_ESM.pdf]

Online resource 3: Output of the analysis

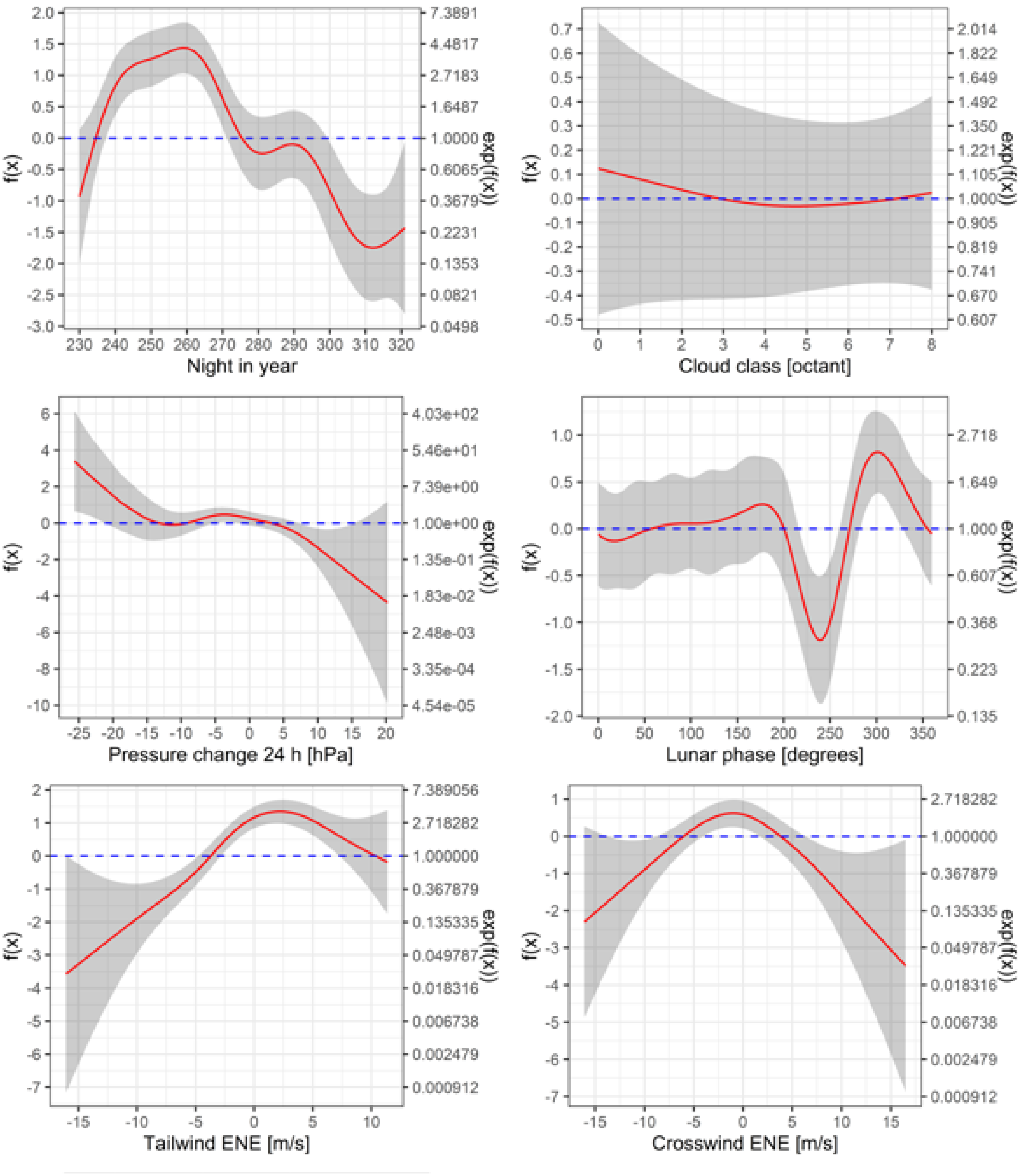

Figure 49. Coefficient plots of the regular smoothers. The plots were made on the linear scale; the y-axis values on the right side show the exponent of the original y-axis values on the left. The solid red lines represents the relation between a covariate value (x-axis) and the effect of the covariate value (y-axis). The ribbon (shaded band) gives the 95% confidence interval. The qualitative interpretation is as follows: higher values of f(x) indicate higher expected probability of detection, lower values indicate lower probability. The quantitative interpretation is as follows: suppose the covariate x changes from value a to value b and all other covariates do not change, then the odds of bat presence will be multiplied by $\frac{\exp(f(x=b))}{\exp(f(x=a))}$.

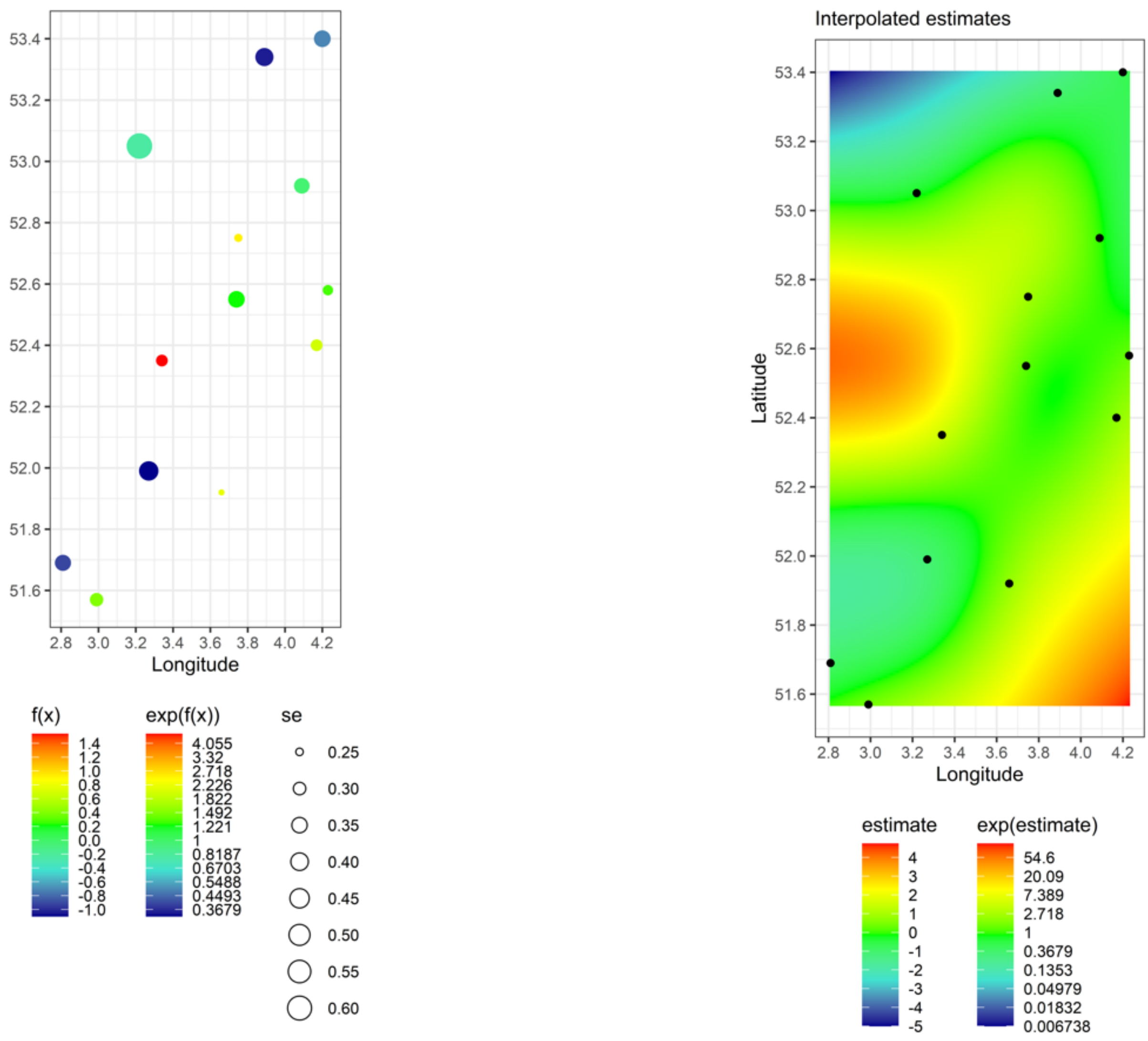

Figure 50. Plot of the longitude-latitude tensor smoother. The plot on the left gives the mean estimate (indicated by colour) and standard error (indicated by the sizes of the points) at the actual monitoring locations. The plot on the right gives the interpolated mean estimates, where the black points indicate the actual monitoring locations. The qualitative interpretation is as follows: higher values of f(x) indicate higher expected probability of detection, lower values indicate lower probability. The qualitative interpretation is as follows: higher values of f(x) indicate higher expected probability of detection, lower values indicate lower probability. The quantitative interpretation is as follows: suppose the spatial coordinates change from coordinates a to coordinates b and all other covariates do not change, then the odds of bat presence will be multiplied by $\frac{\exp(f(\text{coordinate=b}))}{\exp(f(\text{coordinate=a}))}$. Note that the estimates outside the boundaries of the monitoring locations are extrapolated, and thus not reliable.

Table 4: Coefficient summary table of the categorical and linear covariates. The interpretation is as follows: Consider a covariate x; if x increases by a single unit, and all other covariates do not change, than the odds of bat presence is multiplied by exp(β). The exponent of the 95% confidence interval (see columns “exp(q0.025)” and “exp(q0.975)”) is also given in this table.

term	B	Se	exp(β)	exp(q0.025)	exp(q0.975)	p-value
(Intercept)	-3.3772	0.3990	0.0341	0.0156	0.0746	<2.22e-16***
Year 2018	-0.012581	0.291677	0.987498	0.557520	1.749088	0.965596
Year 2019	-0.344998	0.301178	0.708222	0.392470	1.278005	0.252005
Year 2020	-1.309882	0.336650	0.269852	0.139498	0.522015	9.9861e-05***
Rain	-0.8432	0.5030	0.4303	0.1606	1.1532	0.093644
Atmospheric pressure	0.0058	0.0144	1.0058	0.9778	1.0347	0.685948

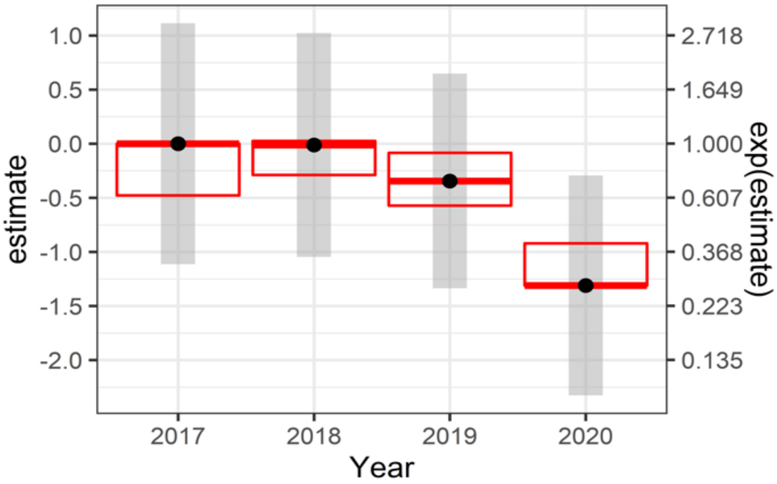

Figure 51. Coefficient estimate plot of the year categories. The plots were made on the linear scale; the y-axis values on the right side show the exponent of the original y-axis values on the left. The black points indicate the correction factor of the intercept (2017 is the reference value, and thus has a value of zero), the grey shades indicate the 95% confidence intervals of the means, and the red crossbars indicate the confidence intervals for the Tukey tests for pairwise differences. If the red crossbars of two categories do not overlap with each other, differences can be regarded statistically significant. The qualitative interpretation is as follows: higher values of f(x) indicate higher expected probability of detection, lower values indicate lower probability. The quantitative interpretation is as follows: suppose the year changes from year a to year b and all other covariates do not change, then the odds of bat presence will be multiplied by $\frac{\exp(f(\text{year}=b))}{\exp(f(\text{year}=a))}$.
